# Plectin promotes tumor formation by B16 mouse melanoma cells via regulation of Rous sarcoma oncogene activity

**DOI:** 10.1186/s12885-022-10033-4

**Published:** 2022-08-30

**Authors:** Kana Mizuta, Takuma Matsubara, Akino Goto, William N. Addison, Mitsushiro Nakatomi, Kou Matsuo, Yukiyo Tada-Shigeyama, Tatsuki Yaginuma, Hiromi Honda, Izumi Yoshioka, Shoichiro Kokabu

**Affiliations:** 1 Division of Molecular Signaling and Biochemistry, Department of Health Improvement, Kyushu Dental University, Kitakyushu, Japan; 2 Division of Oral Medicine, Department of Science of Physical Function, Kyushu Dental University, Kitakyushu, Japan; 3 Department of Human, Information and Life Sciences, School of Health Sciences, University of Occupational and Environmental Health, Kitakyushu, Japan; 4 Division of Oral Pathology, Department of Health Improvement, Kyushu Dental University, Kitakyushu, Japan; 5 Division of Dental Anesthesiology, Department of Science of Physical Function, Kyushu Dental University, Kitakyushu, Japan; 6 Division of Oral and Maxillofacial Surgery, Department of Science and Physical Function, Kyushu Dental University, Kitakyushu, Japan; 7 School of Oral Health Sciences, Kyushu Dental University, Kitakyushu, Japan

**Keywords:** Melanoma, Src, Plectin, Tumor genesis, Cell adhesion

## Abstract

**Background:**

Melanoma is a malignant tumor characterized by high proliferation and aggressive metastasis. To address the molecular mechanisms of the proto-oncogene, Rous sarcoma oncogene (Src), which is highly activated and promotes cell proliferation, migration, adhesion, and metastasis in melanoma. Plectin, a cytoskeletal protein, has recently been identified as a Src-binding protein that regulates Src activity in osteoclasts. Plectin is a candidate biomarker of certain tumors because of its high expression and the target of anti-tumor reagents such as ruthenium pyridinecarbothioamide. The molecular mechanisms by which plectin affects melanoma is still unclear. In this study, we examined the role of plectin in melanoma tumor formation.

**Methods:**

We used CRISPR/Cas9 gene editing to knock-out plectin in B16 mouse melanoma cells. Protein levels of plectin and Src activity were examined by western blotting analysis. In vivo tumor formation was assessed by subcutaneous injection of B16 cells into nude mice and histological analysis performed after 2 weeks by Hematoxylin-Eosin (H&E) staining. Cell proliferation was evaluated by direct cell count, cell counting kit-8 assays, cyclin D1 mRNA expression and Ki-67 immunostaining. Cell aggregation and adhesion were examined by spheroid formation, dispase-based dissociation assay and cell adhesion assays.

**Results:**

In in vivo tumor formation assays, depletion of plectin resulted in low-density tumors with large intercellular spaces. In vitro experiments revealed that plectin-deficient B16 cells exhibit reduced cell proliferation and reduced cell-to-cell adhesion. Since Src activity is reduced in plectin-deficient melanomas, we examined the relationship between plectin and Src signaling. Src overexpression in plectin knockout B16 cells rescued cell proliferation and improved cell-to-cell adhesion and cell to extracellular matrix adhesion.

**Conclusion:**

These results suggest that plectin plays critical roles in tumor formation by promoting cell proliferation and cell-to-cell adhesion through Src signaling activity in melanoma cells.

**Supplementary Information:**

The online version contains supplementary material available at 10.1186/s12885-022-10033-4.

## Background

Melanoma is an aggressive tumor derived from skin melanocytes [1, 2]. Patient prognosis is usually poor because the rapidly proliferating melanoma progresses from radial to vertical growth and frequently metastasizes [1, 2]. Recently developed therapeutic interventions target melanoma metastasis by blocking immune checkpoints using cytotoxic T-lymphocyte-associated antigen 4 (CTLA-4) or programmed death 1 (PD-1) inhibitors [3]. However, these reagents occasionally cause adverse immune-related events such as large intestinal inflammation, interstitial pneumonia, and type I diabetes [4]. Furthermore, some tumors fail to respond to therapy and acquired resistance during immune checkpoint therapy can occur. In addition, melanoma secretes immune suppressing agents such as cortisol and lymphotoxic intermediates [5]. Therefore, novel therapeutic approaches need to be developed.

Rous sarcoma oncogene (Src), a non-receptor tyrosine kinase, plays a physiological role in several cellular processes [6–9] including proliferation, adhesion, migration, and actin filament organization [7, 8]. Src signal transduction is initiated by the phosphorylation of substrates such as protein tyrosine kinase 2 beta (Pyk2) [8, 10] or actin-binding proteins. Src activation suppresses stress fiber formation, leading to the conversion of spindle-shaped cells into round cells with dot-like actin structures [11]. Src, which was originally identified as the first proto-oncogene, is involved in the malignancy, growth, and invasion of several tumors, including melanoma [12–17].

Plectin is a large protein consisting of more than 4000 amino acids. Plectin binds to actin, microtubules, and intermediate filaments, and regulates the cytoskeleton, cell shape, and chromosomal structure [18, 19]. Plectin is also involved in the organization of keratin intermediate filament networks within cells. Disruption of Plectin-linked cytoskeletal networks leads to Src and extracellular signal-regulated kinase (Erk) activation [20]. On the other hand, it has also been demonstrated that plectin is essential for Src activation during osteoclastogenesis [21, 22]. In osteoclasts, plectin acts as a scaffold for the recruitment of Src and Pyk2 substrates [22]. Plectin is a candidate biomarker for certain types of tumors because its expression in tumor specimens is higher than that in surrounding normal tissues [23–25]. Recently, a novel anti-tumor agent targeting plectin, ruthenium pyridinecarbothioamide, was shown to suppress tumor growth in a B16 melanoma cell model [26, 27]. These studies suggest that plectin plays an important role in melanoma pathology and is a good candidate for novel melanoma therapies. However, the relationship between plectin and Src activation in tumor cells is unclear.

In this study, we examined the role of plectin and its interaction with Src signaling in melanoma cells.

## Methods

### Cell culture

B16 (RRID: CVCL_F936) and HMV-II (RRID: CVCL_1282) cells were purchased from RIKEN BRC (Ibaraki, Japan). B16 cells were maintained in Dulbecco’s Modified Eagle Medium (DMEM; Fujifilm Wako, Osaka, Japan) supplemented with 10% fetal bovine serum (fetal bovine serum [FBS]; Nichirei – lot number S.18M00C) [28]. HMVII cells were maintained in Ham’s F12 medium (Fujifilm Wako), supplemented with 10% FBS. HMV-II cells were authenticated by STR (or SNP) profiling by RIKEN BRC. B16 and HMV-II cells were cultured in mycoplasma-free conditions. Cells were transfected using Lipofectamine 2000 (Thermo Fisher Scientific, Waltham, MA, USA), according to the manufacturer’s instructions.

### Establishment of plectin knockout B16 cells and knockdown of plectin in HMVII cells

Mouse plectin-targeting oligonucleotide 5′- GGCGATGTCGGAGATAGTCC − 3′ was cloned into a pGuide-it-ZsGreen1 vector (Takara Bio Inc., Shiga, Japan) and transfected into B16. Single-cell clones were selected by limiting dilution. Plectin expression was determined using western blot analysis [21, 22] .

Human plectin targeting oligonucleotides 5′- GGCGTAGGAAATACAGTTGTGATCTCGAGATCACAACTGTATTTCCTACGTTTTT − 3′ (human plectin shRNA1) and 5′- GGCCTCTTCGATGAGGAGATGAACTCGAGTTCATCTCCTCATCGAAGAGGTTTTT − 3′ (human plectin shRNA2) were cloned into pSIREN-Retro-Q-Zsgreen (Takara Bio Inc.), as described previously [21, 22].

### Western blot analysis

Cells were lysed with a 1% Triton X-containing lysis buffer, as described previously [22]. Lysates were boiled with sodium dodecyl sulfate (SDS) sample buffer for 5 min at 95 °C [22]. Samples were separated on 7.5, 10, 12 and 15% SDS-PAGE gels and transferred to polyvinylidene difluoride (PVDF) membranes. After blocking with 5% bovine serum albumin (BSA), the membranes were incubated with primary antibodies. The antibodies used were anti-Plectin-1 (#12254), anti-Src pY416 (#2101), anti-Pyk2 (#3292), anti-Pyk2 pY402 (#3291), anti-p38 MAPK (#8690), anti-phospho-p38 MAPK (Thr180/Tyr182) (#9211), anti-p44/42 MAPK (Erk1/2) Antibody (#9102), anti-phospho-p44/42 MAPK (Erk1/2) (Thr202/Tyr204) (#4370) (Cell Signaling Technology, CST, Danvers, MA), anti-Src (ab-1) (Merck, Darmstadt, Germany), anti-DDDDK-tag (Flag-tag, Fla-1), anti-GAPDH mAb-horseradish peroxidase-conjugated (HRP)-DirecT (Medical & Biological Laboratories, MBL, Tokyo, Japan), and β-actin (A2228, Sigma Aldrich Chemicals, St. Louis, MO). The membranes were then incubated with HRP-conjugated anti-mouse or anti-rabbit secondary antibodies. Finally, the blots were imaged using a LAS4000 (Fujifilm Wako) with Immobilon ECL Ultra Western HRP Substrate (Merck).

### Immunocytochemical analysis

A total of 1 × 10^4^ cells were plated on a cover glass and cultured for 1 d. Cells were fixed with 3.7% formaldehyde and washed with phosphate buffered saline (PBS). Cells were permeabilized with PBS containing 0.2% Triton X100 and blocked with 5% BSA for 2 h or 4 h. Cells were then incubated with anti-vimentin (E-5, sc-373,717, Santa Cruz Biotechnology, TX), anti-Ki-67 rabbit monoclonal antibody (ab92742, Abcam Cambridge, UK), anti-Flag-tag in 5% BSA at 4 °C overnight. Target proteins were visualized using Alexa 488-conjugated or Alexa 555-conjugated secondary antibodies (Thermo Fisher Scientific). The actin cytoskeleton was visualized using rhodamine phalloidin (Thermo Fisher Scientific). Nuclei were visualized using 4′,6-diamidino-2-phenylindole (DAPI). Slides were imaged using a BZ-X810 microscope (Keyence, Osaka, Japan) and analyzed using ImageJ software (NIH). Cells with a maximum diameter more than 5 times greater than their minor diameter were defined as “elongated cells” [29].

### Animals and tumor injection

Ten-week-old male BALB/cAJcl-nu/nu mice were purchased from CLEA Japan, Inc. (Tokyo, Japan). All mice were used in accordance with the guidelines of the Animal Care and Use Committee of Kyushu Dental University, based on the Animal Research: Reporting of In Vivo Experiments (ARRIVE) guidelines. This study was approved by the Animal Care and Use Committee of Kyushu Dental University (Approval #19–020). The mice were housed under specific pathogen-free conditions. Anesthesia was performed by intraperitoneal injection of an anesthetic agent cocktail (0.3 mg/kg body weight (b.w.) medetomidine, 4.0 mg/kg b.w. midazolam, and 5.0 mg/kg b.w. butorphanol) [30]. Anesthetized mice were injected subcutaneously with 1 × 10^5^ cells in a 100 μL volume [28] After 2 weeks, mice were anesthetized with an anesthetic agent cocktail and perfused with 4% paraformaldehyde in PBS [31]. Tumors were then dissected, measured with calipers, and weighed using an electronic balance scale. Volumes were calculated using the formula width^2^ × length × 0.52, as previously described [32, 33]. Sections of 10 μm were prepared using a cryostat. The sections were stained with hematoxylin and eosin (H&E) and imaged using a BZ-X810 microscope. Cell numbers and tumor areas were quantified with ImageJ software. Cell density was calculated as the number of the cells divided by tumor area. The area between cells was determined by subtracting total cell area from total tumor area on the histological section. Sections were also stained with 0.1% azocarmine G solution, aniline blue and orange G solution (Azan staining) and imaged using a BZ-X810 microscope.

### Cell proliferation and viability assays

Cell proliferation was assessed with the Cell Counting Kit-8 (CCK-8, Dojindo, Kumamoto, Japan), according to the manufacturer’s protocol [28]. Cells were plated at a density of 5000 cells/well in 96-well plates. For direct viable cell counts by the trypan blue pigment exclusion method, cells were plated at a density of 2.5 × 10^4^ cells/well in 24-well plates [34]. Three days later, cells were detached with trypsin (Fujifilm Wako) mixed with Trypan Blue counted with a TC20 Automated Cell Counter (Bio-Rad).

### Reverse transcription and quantitative PCR (qPCR)

Total RNA was isolated using the FastGene™ RNA Basic Kit (Nippon Genetics, Tokyo, Japan) and reverse-transcribed into cDNA using the High-Capacity cDNA Reverse Transcription Kit (Thermo Fisher Scientific) [35]. Real time quantitative (q)PCR was performed using a Quantstudio 3 system (Thermo Fisher Scientific) with specific primers for murine Cyclin D1 (*Ccnd1*); (forward, 5′- tttctttccagagtcatcaagtgt − 3′; 5′- tgactccagaagggcttcaa − 3′), human Cyclin D1 (*CCND1*); (forward, 5′- tctacaccgacaactccatccg − 3′; 5′- tctggcattttggagaggaagtg − 3′), murine β-actin (*Actb*); (forward, 5′- aaggccaaccgtgaaaagat − 3′; reverse, 5′- gtggtacgaccagaggcatac − 3′), and human β-actin (*ACTB*); (forward, 5′- caccattggcaatgagcggttc − 3′; reverse, 5′- aggtctttgcggatgtccacgt − 3′) [36, 37]. Gene expression levels were calculated using the ΔΔCT method and normalized to that of β-actin.

### Spheroid formation

Cells were seeded at a density of 5000 cells in 15 μL of media on the inner side of a dish lid. The lid was then placed upside down on a PBS-filled plate. Cells were cultured for 7 d at 37 °C, with 5% CO_2_. Images of spheroids were taken with a Leica EZ4 HD microscope (Leica, Germany) and analyzed using ImageJ software. The volumes were calculated using the formula width^2^ × length × 0.52, similar to that used for the tumor.

### Dispase-based dissociation assay

Cells were seeded at a density of 1.0 × 10^6^ cells/well in 6-well plates. Twenty-four hours after confluency, the cells were incubated with 1440 PU/ml dispaseII (Fujifilm Wako) solution for 40 min at 37 °C to detach the monolayer from the well bottom. The dispase solution was then carefully transferred into a new 6-well plate using a 1 ml pipette. Next, the cell monolayer was mechanically stressed by pipetting up and down 7 times [38, 39].

### Cell adhesion assay

96-well plates were coated by overnight incubation with 10 μg/mL fibronectin at 4 °C. Wells were then blocked with 2% BSA for 2 h at room temperature (RT). Cells were plated at a density of 3 × 10^4^ cells per well in serum-free medium and allowed to attach for 2 h at 37 °C with 5% CO_2_. Non-adherent cells were washed away with serum-free medium. The remaining adherent cells were fixed using 1% glutaraldehyde/PBS for 10 min and stained with crystal violet in 2% methanol/PBS for 20 min at RT with shaking. Crystal violet was solubilized with 1% SDS and quantified by absorbance measurement at 595 nm using a microplate reader (Bio-Rad, Hercules, CA). Adherent cells were counted using ImageJ software.

### Src overexpression

Src mutating tyrosine 527 to phenylalanine (constitutively activated Src) with a Flag-tag was previously described [40, 41]. Constitutively activated Src was transfected B16 cells with Lipofectamine 2000 (Thermo Fisher Scientific) and cultured for 1 d. Then, cells were seeded and performed each experiment.

### Data analysis and statistics

Statistical significance of differences between groups was analyzed using one-way ANOVA followed by a Tukey-Kramer post-hoc test. Statistical significance was set at *p* < 0.05. Data are expressed as mean ± standard deviation of the mean (n = number of samples). All experiments were independently performed at least twice and similar results were obtained.

### Microarray data mining

Dataset GDS1375 from the NCBI Gene Expression Omnibus (GEO) was analyzed for plectin and Src gene expression in melanoma patient samples, as previously described [28, 42]. Means were compared using Kruskal-Wallis followed by Dunn’s post hoc test.

## Results

### Src signaling is impaired in plectin-deficient melanoma cells

**Fig. 1:**
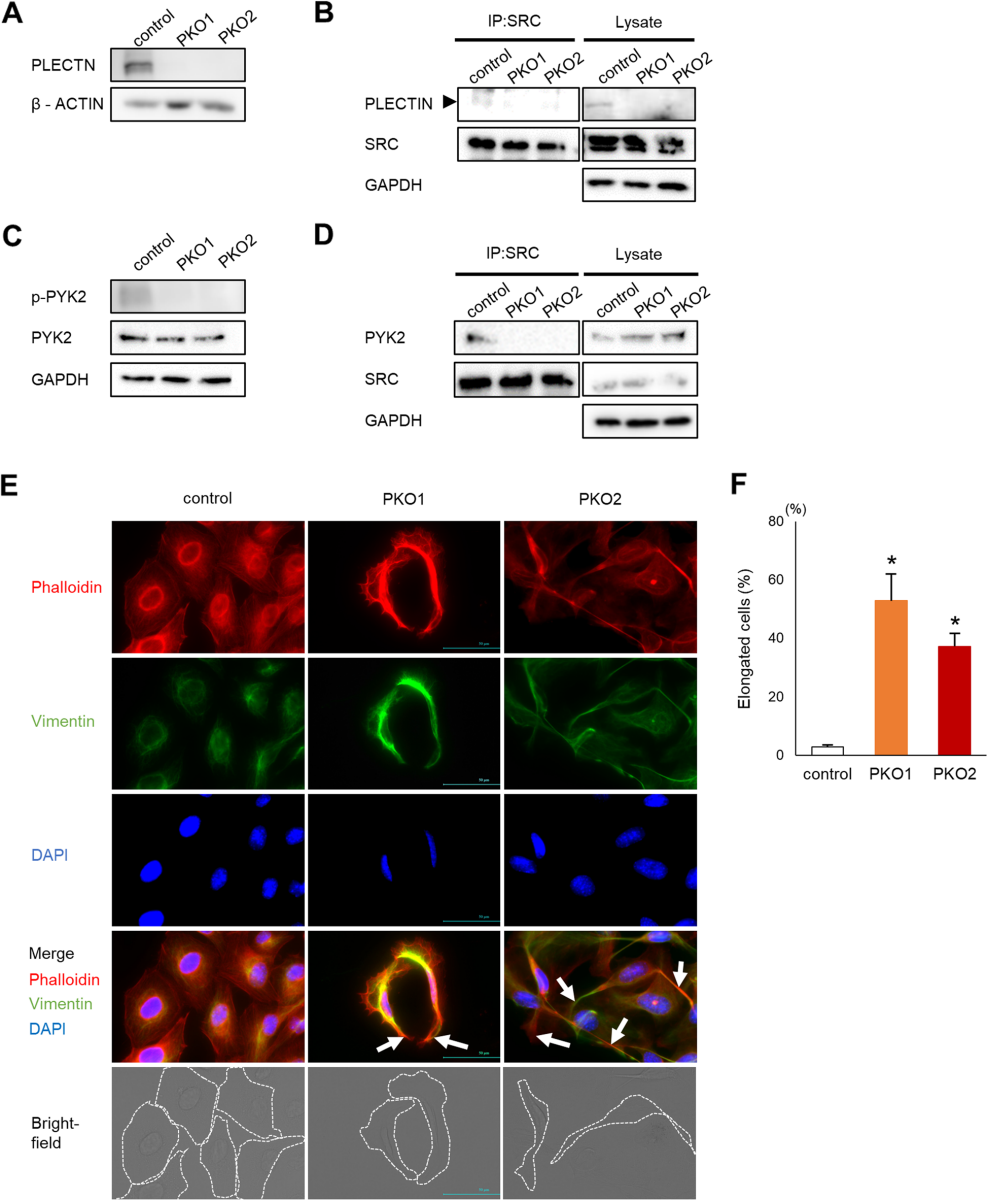
Src signaling is impaired in plectin deficient cells. **A** PLECTIN levels in control B16 and 2 clones of plectin deficient B16 cells (PKO1 and PKO2) were determined by western blotting analysis. **B** SRC was immunoprecipitated with an anti-Src antibody (IP:SRC) and interaction with PLECTIN was detected by western blotting analysis. **C** Expression and phosphorylation of PYK2, or GAPDH was determined by western blotting assay 24 h after plating. **D** Src was immunoprecipitated with an anti-Src antibody (IP:SRC) and interaction with PYK2 was detected by western blotting analysis. **E** Cells were stained with Rhodamine-phalloidin and vimentin/anti-mouse Alexa fluor 488. Long actin fibers are indicated by arrows. Cell shapes are outlined with a dotted line in the bright-field image. Scale bar = 50 μm. **F** Quantification of the number of elongated cells in samples shown in (**E**) (*n* = 5). *; *p* < 0.05 vs control

To examine the role of plectin in melanoma, we used CRISPR/Cas9 gene editing to delete plectin in murine B16 melanoma cells (PKO cells). Western blotting confirmed successful deletion of plectin (Fig. 1A). We have previously shown that in osteoclast cells plectin interacts with Src and facilitates Src activation [22]. We next examined whether plectin also interacts with src in melanoma cells. By co-immunoprecipitation, we confirmed that plectin and Src are bound in control wildtype B16 cells but not in plectin knockout cells (Fig. 1B). Furthermore, Src activation, determined by the level of phospho-Pyk2 (Tyr402), was decreased in plectin-deficient cells (Fig. 1C). Moreover, binding of Src to Pyk2 was also disrupted in PKO cells (Fig. 1D). Spindle-shaped cells with thick, long actin fibers and abnormal cell spreading are characteristic of disturbed Src signaling [29]. Consistent with abnormal Src activity in PKO cells, we observed that PKO cells were elongated and less round than control cells (Fig. 1E, F). Vimentin, an intermediate fiber regulated by plectin, forms networks around the nuclei in control B16 cells. In contrast, vimentin localization was diffused in plectin deficient cells (Fig. 1E and F).

To determine whether these observations were cell-type specific, we also generated plectin deficient human HMV-II human melanoma cells by shRNA mediated knockdown (Fig. S1A). Similar to our observations in B16 cells, plectin knockdown reduced Src signaling (Fig. S1B) and produced cells with abnormal elongated morphology (Figs. S1C and S1D). Thus, the depletion of plectin impairs Src signaling in melanoma cells.

### Plectin regulates cell proliferation and adhesion

**Fig. 2:**
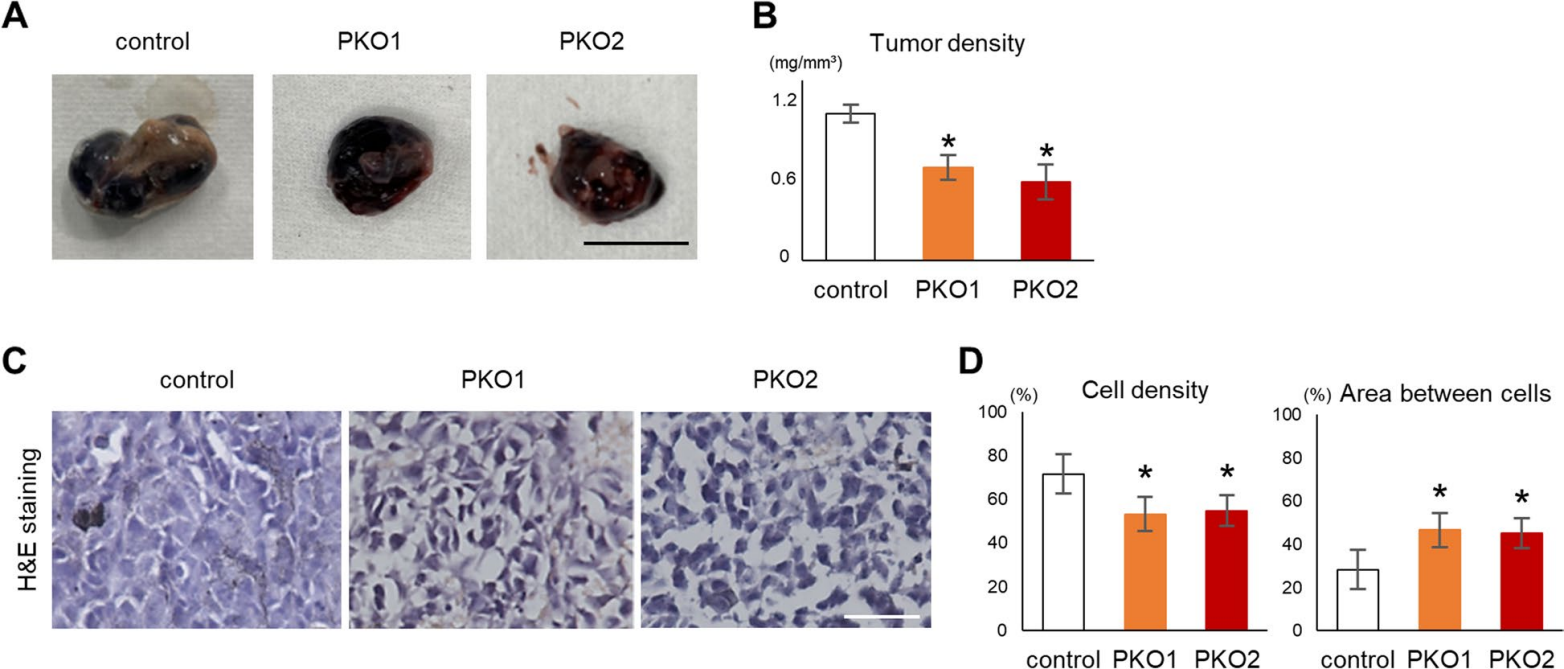
Plectin knockout cells form low density tumors. **A** Control or PKO cells (1 × 10^5^ cells per mouse) were subcutaneously injected into BALB/cAJcl-nu/nu mice (*n* = 8). Photographs of extracted tumors 2 weeks after injection. Scale bar = 10 mm. **B** Tumor density was calculated from excised tumor weight and volume. Averages of the total tumor density of all tumors excised from each mouse is shown in the graph (*n* = 8). **C** Sections of tumors were stained with H&E. Scale bar = 50 μm. **D** Cell density and the area between cells were calculated. (*; *p* < 0.05 vs control)

To determine the function of plectin in tumor formation in vivo, control or PKO cells were subcutaneously injected into the backs of nude mice (Fig. 2A). There were no significant differences in tumor weight or volume between control and PKO cells (Figs. S2A and S2B). However, the density of PKO tumors was lower than that of the control tumors (Fig. 2B). Histological analysis showed that the area between cells and subsequently the cell density within the PKO tumor was greater than in control tumors (Fig. 2C and D). Azan staining, which usually stains collagen and extracellular matrix blue, showed that the intercellular spaces did not contain any collagenous extracellular matrix (Fig. S2C). No other extracellular elements other than some red blood cells were detected in the tumors. Thus, intercellular spaces may be filled with interstitial fluid similar to edemas or the spontaneous skin blistering that occurs in patients with plectin mutations who develop epidermolysis bullosa simplex [43–45]. Taken together, these in vivo data suggest that plectin may affect cell number and intercellular connections.

**Fig. 3:**
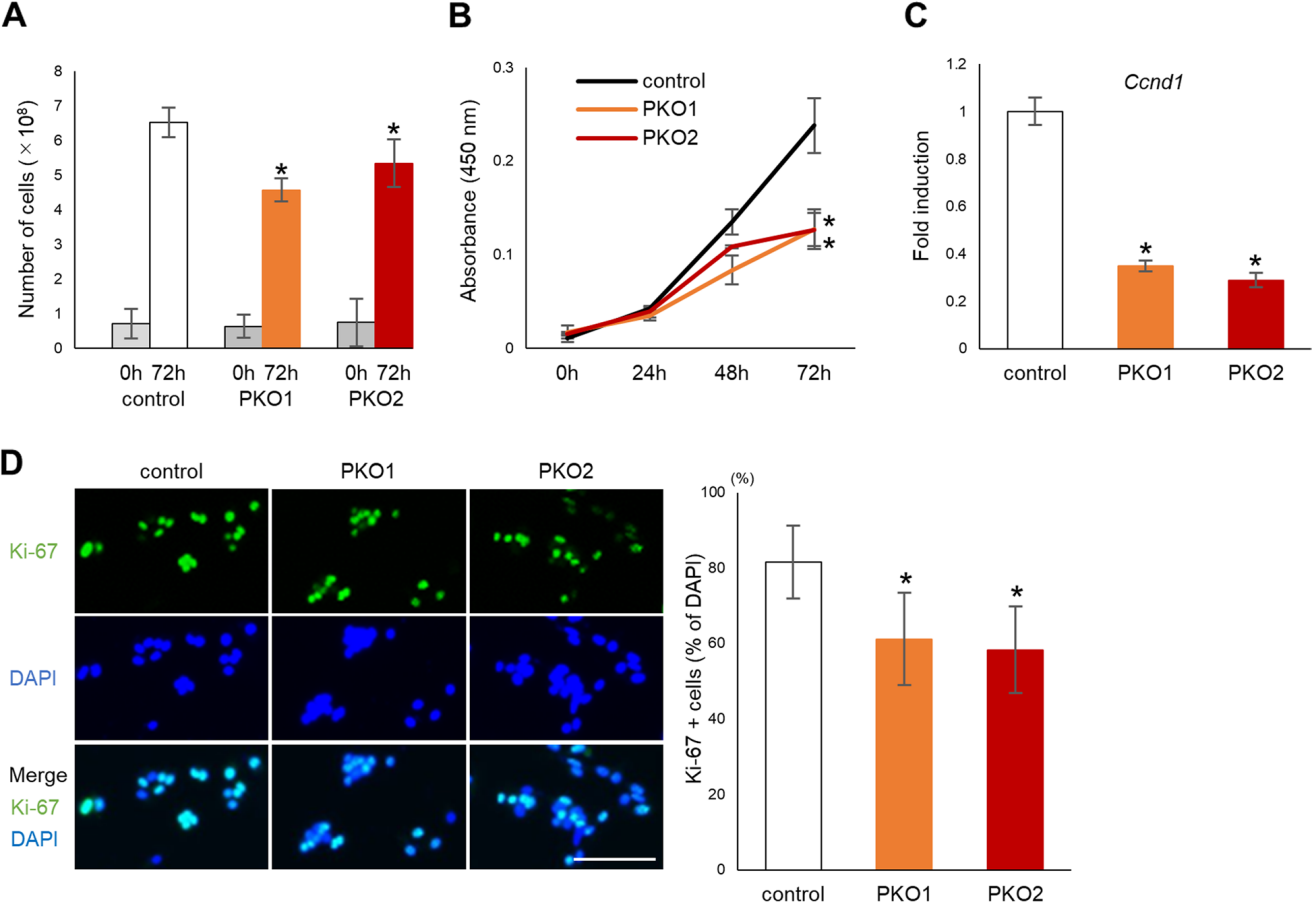
Cell proliferation is decreased in plectin knockout cells. **A** Cells were plated at a density of 2.5 × 10^4^ cells per well in 24-well plates for the indicated time period after which cells were trypsinized and the number of cells determined with an automated cell counter (*n* = 6). **B** Cells were plated at a density of 5 × 10^3^ cells per well in a 96 well plate and the number of viable cells measured by CCK-8 assay at the indicated time (*n* = 4). **C** mRNA was harvested from control or PKO cells 1 d after plating. Expression of *Ccnd1* was determined by real time qPCR (*n* = 3). **D** Control or plectin knockout B16 cells were fixed and stained with anti-Ki-67/Alexa fluor 555 and DAPI. The percentage of Ki-67 positive cells relative to DAPI positive cells was calculated. (*n* = 5), *; *p* < 0.05 vs control

Next, the effects of plectin on cell proliferation was examined in vitro. Direct cell counting as well as a CCK-8 assay showed that the number of viable cells were significantly reduced after plectin depletion in B16 cells (Fig. 3A and B) or in HMV-II cells (Figs. S3A and S3B). mRNA levels of Cyclin D1, a regulator of cell cycle progression, were also decreased in plectin-deficient cells (Figs. 3C and S3C). Immunofluorescence analysis showed that the number of Ki-67 positive cells, a marker of cell proliferation, was also decreased in plectin-deficient cells (Figs. 3D and S3D).

**Fig. 4:**
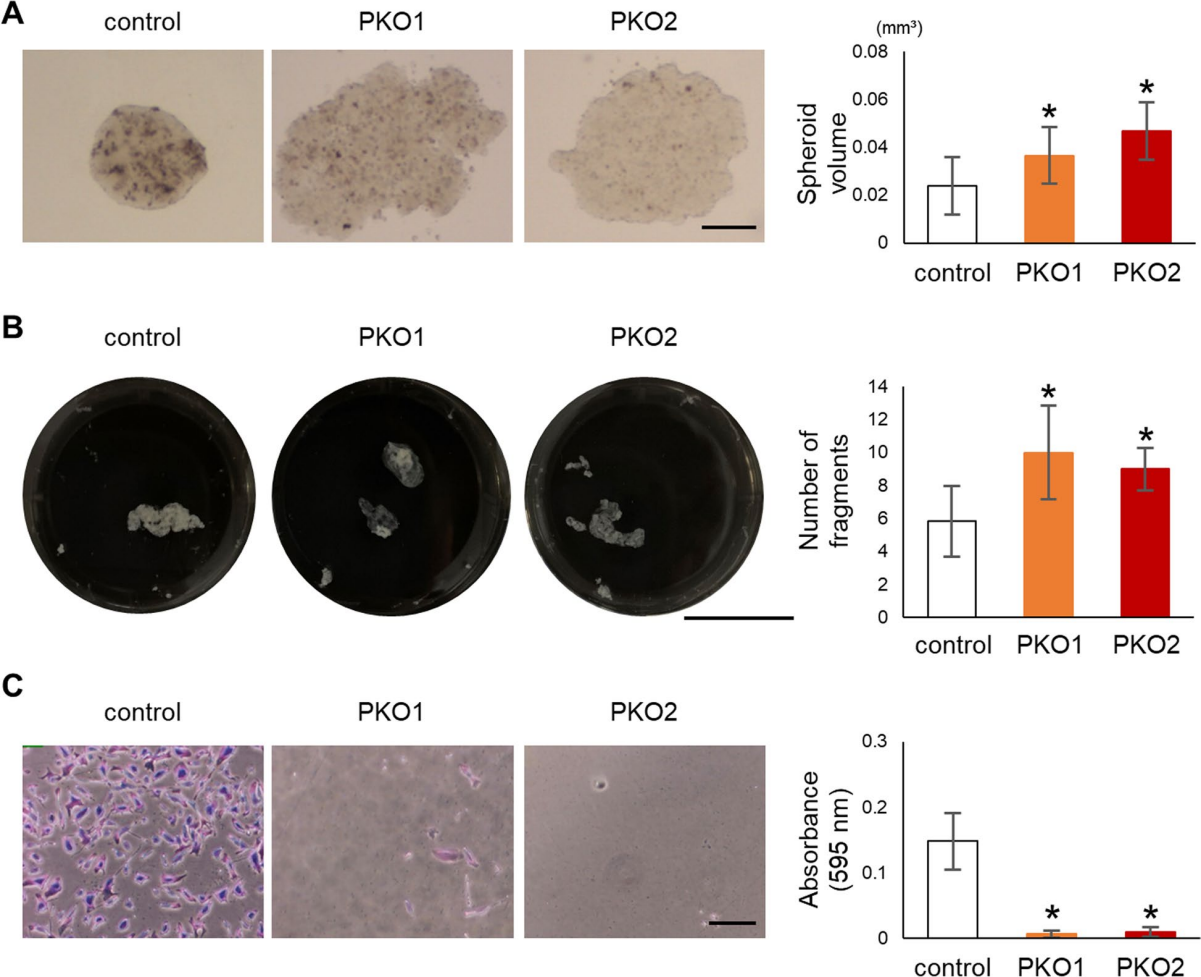
Cell adhesion is impaired in plectin knockout cells. **A** Cells were cultured at a density of 5 × 10^3^ cells per 15 μl drop on the inner side of a dish lid to form spheroids for 7 d. Images were acquired with a stereoscopic microscope and volumes quantified with ImageJ. Scale bar = 250 μm (*n* = 5). **B** Confluent cells were detached with dispase and mechanically fragmented by pipetting. The left panel shows an image of the fragmented cell monolayer and the right panel shows quantification of the number of cell fragments. Scale bar = 20 mm (*n* = 6). **C** Cells were plated on a fibronectin-coated dish and counted the number of adherent cells after 2 h. The adherent cells were stained with crystal violet (left 3 panels). After taken photo, the adherent cells were lysed and measured the absorbance shown at graph (right panel) (*n* = 6), *; *p* < 0.05 vs control

To determine the role of plectin in the formation of intercellular cell connections we next performed a spheroid formation assay using PKO cells. Similar to our observation in tumor formation in vivo, plectin-deficient cells formed larger spheroids than the control cells (Figs. 4A and S4A). A dispase-based dissociation assay showed that PKO cell monolayers were more sensitive to mechanical fragmentation indicative of weakened cell-to-cell adhesion (Fig. 4B). In addition, cell attachment to fibronectin was reduced in plectin-deficient cells compared to that in control cells in an adhesion assay (Figs. 4C and S4B). This suggests that plectin plays an important role in both cell proliferation and in the formation of cell-to-cell or cell-to-matrix adhesion of melanoma cells.

### Overexpression of Src rescues the plectin knockout phenotype

**Fig. 5:**
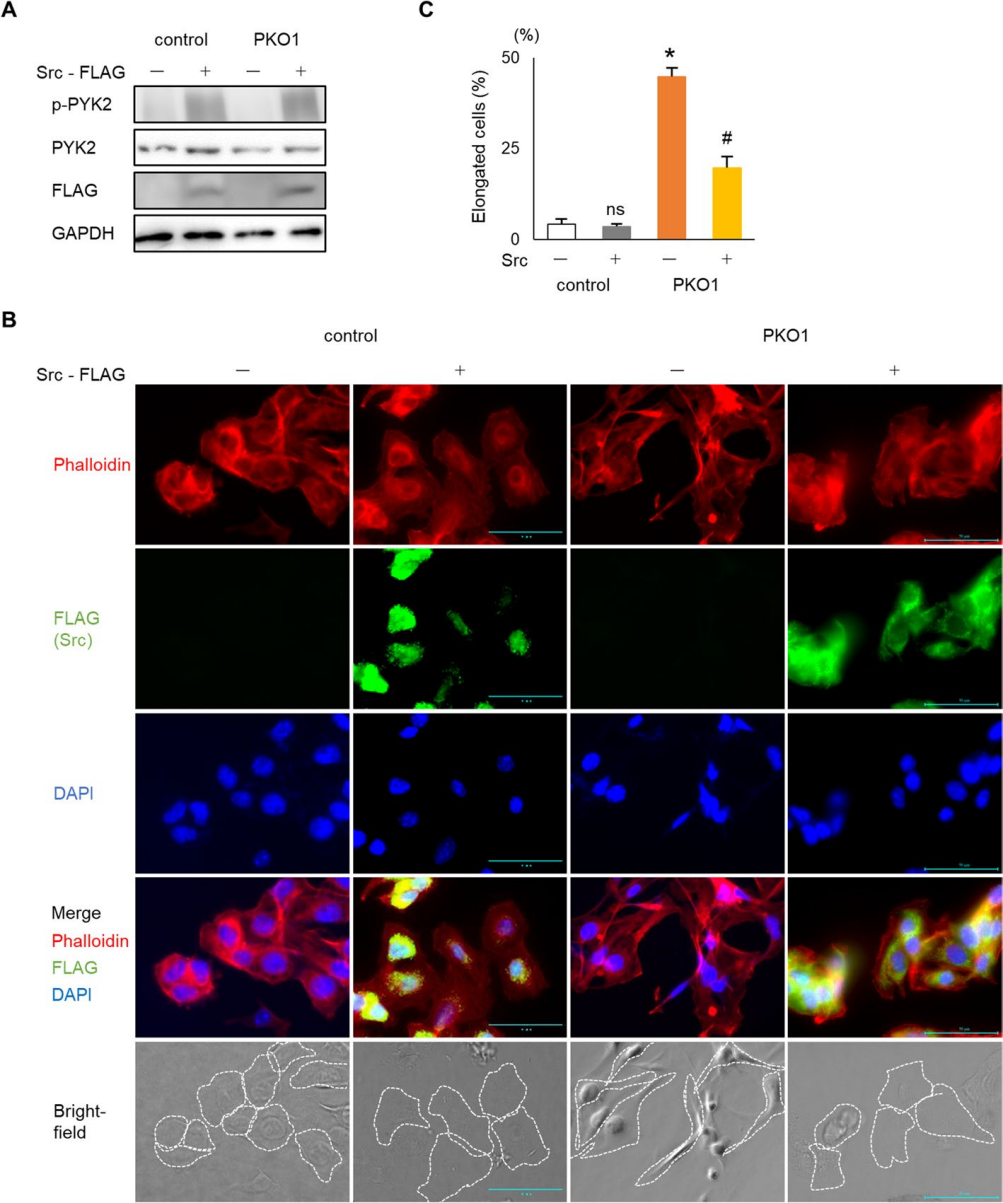
Src overexpression alters cell shape of plectin knockout melanoma cells. **A** Src was overexpressed in B16 and PKO1. Expression and phosphorylation of PYK2 was determined by western blotting analysis. **B** Cells were immunostained with anti-Flag/Alexa fluor 488, Rhodamine-phalloidin and DAPI 1 d after Src introduction. Cell shapes are outlined with a dotted line in the bright-field image. Scale bar = 50 μm. **C** The number of elongated cells were quantified (*n* = 5). *; *p* < 0.05 vs control Src(−), #; *p* < 0.05 vs PKO1 Src(−), ns; no significance vs control Src(−)

**Fig. 6:**
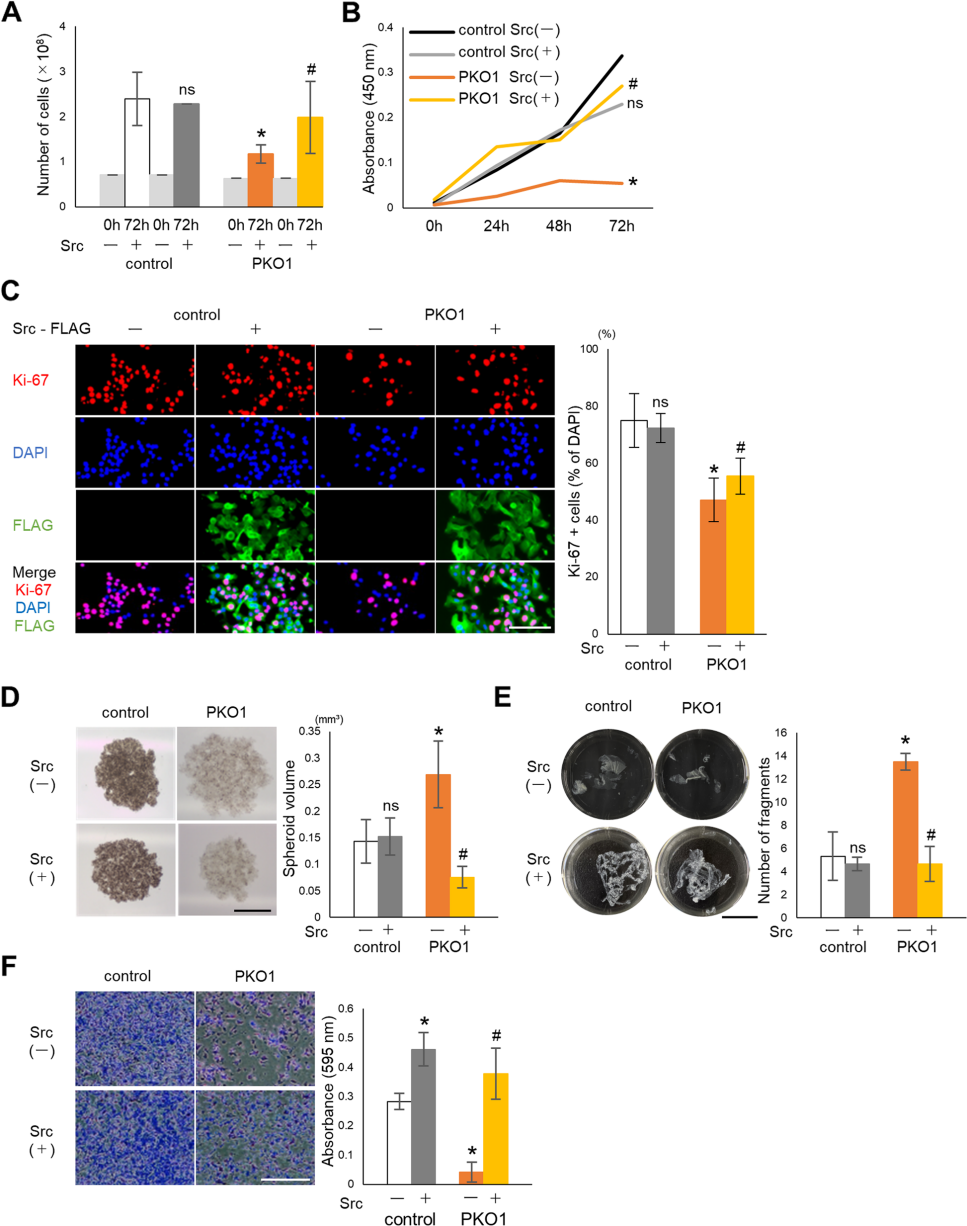
Src overexpression rescues cell proliferation and adhesion of plectin knockout melanoma cells. **A** Cells were plated at a density of 2.5 × 10^4^ cells per well in 24-well plates and the number of cells quantified with an automated cell counter (*n* = 6). **B** Cells were plated at 1 d after Src introduction. The number of viable cells were determined by CCK-8 assay at indicated time (*n* = 3). **C** Cells were fixed and stained with DAPI, anti-Ki-67/Alexa fluor 555 and anti-Flag/Alexa fluor 488 at 1 d after plating. The percentage of Ki-67 positive cells relative to DAPI was calculated. Scale bur = 100 μm (*n* = 5). **D** Src was introduced into the cells for 1 d. Cells were cultured at 5 × 10^3^ cells per 15 μl drop on a dish lid to form spheroid for 7 d. Spheroids were observed by stereoscopic microscope and measured by ImageJ. Scale bar = 500 μm (*n* = 6). **E** The confluent cells were detached with dispase and number of cell fragments was taken photo (left panel) and quantified (right panel). Scale bar = 20 mm (*n* = 6). **F** Cells were plated on a fibronectin-coated dish 1 d after Src introduction. After 2 h incubation, the adherent cells were stained with crystal violet (left 4 panels) and counted (right panel). Scale bar = 10 μm (*n* = 6), *; *p* < 0.05 vs control Src(−), #; *p* < 0.05 vs PKO Src(−), ns; no significance vs control Src(−)

Given that Src activity is reduced in PKO cells, we next sought to determine whether plectin affects melanoma cell behavior via Src signaling. We therefore examined the effect of overexpressing a constitutively activated Src construct in PKO cells. Src overexpression was able to increase p-Pyk2 levels in wildtype and PKO cells (Fig. 5A). Overexpression of Src in PKO cells reduced the abnormally high percentage of elongated cells occurring in PKO cell cultures (Fig. 5B and C). In addition, Src overexpression restored the vimentin network in PKO cells to a peri-nuclear localization (Fig. S5). Overexpression of Src also prevented the decreased cell proliferation in PKO cells as determined by the number of viable cells and Ki-67 positive cells (Fig. 6A, B and C). In the spheroid formation assay, Src overexpression in PKO cells led to the formation of small, tightly packed spheroids similar to control cells instead of the large loose phenotype observed in PKO cells without Src overexpression (Fig. 6D). In the dispase-dissociation assay, Src overexpression was able to prevent the fragmentation of PKO cells suggesting that the loss of cell-cell adhesion in PKO cells is Src-dependent (Fig. 6E). Furthermore, cell adhesion to fibronectin was also improved when Src was overexpressed in PKO cells (Fig. 6F).

## Discussion

In this study, we demonstrated that plectin was essential for Src activation in melanoma cells. This is similar to our previous observations in osteoclasts [22]. In plectin-deficient melanomas, proliferation and adhesion are impaired. Overexpression of constitutively activated Src partially rescued the defective proliferation and adhesions suggesting that plectin regulates the proliferation and adhesion of melanoma cells through Src-dependent and Src-independent mechanisms.

The relationship between plectin and Src activation appears to be complex and cell-type dependent. For example, although plectin negatively regulates Src in keratinocytes [20], plectin plays an essential role in activating Src signaling to organize the cytoskeleton in osteoclasts [22]. In the focal adhesion complex, plectin participates in mechanosensing to activate Src signaling downstream of the vimentin intermediate filament network [46]. Our data confirmed that plectin interacts with Src in a manner that promotes cell adhesion similar to our previous observations in osteoclasts.

In tumor cell proliferation, Src mediates growth factor signaling by activating Ras GTPase and mitogen-activated protein kinases (MAPKs) [8]. Src and cyclin-dependent kinase 1 (Cdk1) have common substrates related to cell proliferation [47, 48]. Plectin is known to be involved in cell proliferation independently of Src. Cdk1 interacts with plectin and regulates the rearrangement of the cytoskeleton during interphase to promote mitosis [19]. Plectin directly binds and organizes actin, microtubules, and intermediate filament networks at each step of the cell cycle [19]. Therefore, cell division is disrupted in the absence of plectin.

Src is a major regulator of both cell adhesion and aggregation. Src signaling promotes focal adhesion formation via Pyk2 and Rho GTPase activation for adhesion to the extracellular matrix [49–51]. Plectin has been shown to directly organize vimentin networks, cell polarization and focal adhesions for cell adhesion in fibroblasts [46, 52]. We observed disturbed vimentin network organization in plectin deficient cells that could be restored by Src activation. Src directly phosphorylates and organizes vimentin around the nuclei [53]. Plectin may thus likewise be critical for vimentin organization through its Src dependent functions. Src-independent regulation of vimentin or actin networks by plectin can however not be excluded. Src and Pky2 signaling also regulates cell-to-cell contacts by phosphorylating cadherins during desmosome and tight junction formation [54, 55]. Similarly, plectin also directly organizes the keratin network or vimentin network to maintain tight junction and adherence junction [39, 56–58]. Plectin’s role in the cytoskeletal integrity and cell-cell contacts is evident in human patients with plectin mutations where disruption of keratin networks within the skin leads to extensive blistering or epidermolysis bullosa simplex [43–45].

P38 MAPK and extracellular signal-regulated kinase (Erk) 1/2 have been shown to be the mediators of plectin function in cytoskeleton organization [20, 46]. In our study we found that phosphorylation of p38 and Erk was enhanced in PKO cells (Fig. S6) similar to the plectin-deficient keratinocytes [20]. In melanoma, Erk and p38 are mutually activated and induce cell migration [59]. Erk activation also causes ECM degradation and decreased Cadherin expression [60]. Elevated Erk activation could contribute to the weakened cell adhesion in plectin deficient B16 cells. In keratinocytes, Src increases Erk phosphorylation, while in melanomas, Erk phosphorylation is increased despite low Src activity. Further studies are needed to reveal the extent and mechanisms of MAPK regulation by plectin in the cytoskeleton.

Analysis of a GEO dataset showed that plectin-and Src-related genes are highly expressed in human primary melanoma in patients compared to human melanocytes (Fig. S7). Based on our experimental data, plectin may be required for malignancy and primary tumor formation in melanoma. During metastasis, plectin may play a positive role in the invasion and migration into the vascular system. Src signaling and plectin are essential for organizing actin structures in invadosomes [61, 62]. Plectin may also be necessary for cell anchoring to the target tissue during metastasis or separation of cells from the primary tumor mass. Therefore, the role of plectin in melanoma progression may differ at each phase.

An anti-tumor agent targeting plectin, ruthenium pyridinecarbothioamide, has been recently developed for the treatment of melanoma [26, 27]. Our present study strongly supports a role for plectin in melanoma during early stage events, such as primary tumor formation. However, the effects of plectin inhibition during the late stage of melanoma remain unclear. Furthermore, data from the Human Protein Atlas (https://www.proteinatlas.org/ENSG00000178209-PLEC/pathology/melanoma) and The Cancer Genome Atlas (TCGA) showed that melanoma patients with high plectin expression survived significantly longer than those with low expression levels. This suggests that the suppression of plectin may promote melanoma after the formation of the primary tumor because the primary tumor had already been formed upon diagnosis. Further studies, especially on invasion and metastasis, are required to clarify the role of plectin in melanoma progression.

## Conclusions

Plectin plays a key role in robust tumor formation in melanoma by regulating cell proliferation and aggregation through Src signaling.

## Supplementary Information

**Additional file 1: Fig S1**. Generation of plectin knockdown human melanoma cells. **Fig S2**. Tumor weights and volumes. **Fig S3**. Cell proliferation is decreased in plectin knockdown human melanoma cells. **Fig S4**. Cell adhesion is impaired in plectin knock downed human melanoma cells. **Fig S5**. Vimentin was localized around the nucleus following Src activation in PKO cells. **Fig S6**. Plectin knockout decreased MAPK signaling. **Fig S7**. Expression of plectin and SRC-related genes are increased in human primary melanoma samples.

**Additional file 2.**

## Data Availability

The datasets used and/or analyzed during the current study are available from the corresponding author on reasonable request. The dataset in Fig. S5 is available from GSE1375 dataset (GEO accession number 1375) of NCBI Gene Expression Omnibus (GEO) (https://www.ncbi.nlm.nih.gov/geo/query/acc.cgi?acc=GSE1375).

## References

[1] Rigel DS, Carucci JA (2000). Malignant melanoma: prevention, early detection, and treatment in the 21^st^ century. CA Cancer J Clin.

[2] Perera E, Gnaneswaran N, Jennens R, Sinclair R (2013). Malignant Melanoma. Healthcare (Basel).

[3] Larkin J, Hodi FS, Wolchok JD (2015). Combined nivolumab and ipilimumab or monotherapy in untreated melanoma. N Engl J Med.

[4] Horvat TZ, Adel NG, Dang T-O, Momtaz P, Postow MA, Callahan MK (2015). Immune-related adverse events, need for systemic immunosuppression, and effects on survival and time to treatment failure in patients with melanoma treated with ipilimumab at memorial Sloan Kettering cancer center. J Clin Oncol.

[5] Slominski AT, Carlson JA (2014). Melanoma resistance: a bright future for academicians and a challenge for patient advocates. Mayo Clin Proc.

[6] Soriano P, Montgomery C, Geske R, Bradley A (1991). Targeted disruption of the c-src proto-oncogene leads to osteopetrosis in mice. Cell..

[7] Vara JÁF, Cáceres MAD, Silva A, Martín-Pérez J. (2001). Src family kinases are required for prolactin induction of cell proliferation. Mol Biol Cell.

[8] Kim LC, Song L, Haura EB (2009). Src kinases as therapeutic targets for cancer. Nat Rev Clin Oncol.

[9] Okada M (2012). Regulation of the SRC family kinases by Csk. Int J Biol Sci.

[10] Zhao M, Finlay D, Zharkikh I, Vuori K (2016). Novel role of Src in priming Pyk2 phosphorylation. PLoS One.

[11] Berdeaux RL, Díaz B, Kim L, Martin GS (2004). Active Rho is localized to podosomes induced by oncogenic Src and is required for their assembly and function. J Cell Biol.

[12] Irby RB, Yeatman TJ (2000). Role of Src expression and activation in human cancer. Oncogene..

[13] Qi J, Wang J, Romanyuk O, Siu C-H (2006). Involvement of Src family kinases in N-cadherin phosphorylation and beta-catenin dissociation during transendothelial migration of melanoma cells. Mol Biol Cell.

[14] Eustace AJ, Crown J, Clynes M, O’Donovan N (2008). Preclinical evaluation of dasatinib, a potent Src kinase inhibitor, in melanoma cell lines. J Transl Med.

[15] Oneyama C, Hikita T, Nada S, Okada M (2008). Functional dissection of transformation by c-Src and v-Src. Genes Cells.

[16] Homsi J, Cubitt CL, Zhang S, Munster PN, Yu H, Sullivan DM (2009). Src activation in melanoma and Src inhibitors as therapeutic agents in melanoma. Melanoma Res.

[17] Sánchez-Bailón MP, Calcabrini A, Gómez-Domínguez D, Morte B, Martín-Forero E, Gómez-López G (2012). Src kinases catalytic activity regulates proliferation, migration and invasiveness of MDA-MB-231 breast cancer cells. Cell Signal.

[18] Andrä K, Lassmann H, Bittner R, Shorny S, Fässler R, Propst F (1997). Targeted inactivation of plectin reveals essential function in maintaining the integrity of skin, muscle, and heart cytoarchitecture. Genes Dev.

[19] Steinböck FA, Wiche G (1999). Plectin: a cytolinker by design. Biol Chem.

[20] Osmanagic-Myers S, Gregor M, Walko G, Burgstaller G, Reipert S, Wiche G (2006). Plectin-controlled keratin cytoarchitecture affects MAP kinases involved in cellular stress response and migration. J Cell Biol.

[21] Matsubara T, Kinbara M, Maeda T, Yoshizawa M, Kokabu S, Takano YT (2017). Regulation of osteoclast differentiation and actin ring formation by the cytolinker protein plectin. Biochem Biophys Res Commun.

[22] Matsubara T, Yaginuma T, Addison WN, Fujita Y, Watanabe K, Yoshioka I (2020). Plectin stabilizes microtubules during osteoclastic bone resorption by acting as a scaffold for Src and Pyk2. Bone..

[23] Bausch D, Thomas S, Mino-Kenudson M, Fernández-del CC, Bauer TW, Williams M (2011). Plectin-1 as a novel biomarker for pancreatic cancer. Clin Cancer Res.

[24] Katada K, Tomonaga T, Satoh M, Matsushita K, Tonoike Y, Kodera Y (2012). Plectin promotes migration and invasion of cancer cells and is a novel prognostic marker for head and neck squamous cell carcinoma. J Proteomics.

[25] Tamura I, Ueda K, Nishikawa T, Kamada A, Okamura T, Matsuda Y (2018). Expression of Plectin-1 and Trichohyalin in human tongue Cancer cells. OJST..

[26] Meier SM, Kreutz D, Winter L, Klose MHM, Cseh K, Weiss T (2017). An organoruthenium anticancer agent shows unexpected target selectivity for plectin. Angew Chem Int Ed Engl.

[27] Klose MHM, Schöberl A, Heffeter P, Berger W, Hartinger CG, Koellensperger G (2018). Serum-binding properties of isosteric ruthenium and osmium anticancer agents elucidated by SEC-ICP-MS. Monatsh Chem.

[28] Ogawa M, Yaginuma T, Nakatomi C, Nakajima T, Tada-Shigeyama Y, Addison WN (2019). Transducin-like enhancer of split 3 regulates proliferation of melanoma cells via histone deacetylase activity. Oncotarget..

[29] Katoh K. Regulation of fibroblast cell polarity by Src tyrosine kinase. Biomedicines. 2021;9:135. 10.3390/biomedicines9020135 PMC7912711 33535441

[30] Kirihara Y, Takechi M, Kurosaki K, Kobayashi Y, Kurosawa T (2013). Anesthetic effects of a mixture of medetomidine, midazolam and butorphanol in two strains of mice. Exp Anim.

[31] Nakanishi M, Hata K, Nagayama T, Sakurai T, Nishisho T, Wakabayashi H (2010). Acid activation of Trpv1 leads to an up-regulation of calcitonin gene-related peptide expression in dorsal root ganglion neurons via the CaMK-CREB cascade: a potential mechanism of inflammatory pain. Mol Biol Cell.

[32] Tada Y, Kokabu S, Sugiyama G, Nakatomi C, Aoki K, Fukushima H (2014). The novel IκB kinase β inhibitor IMD-0560 prevents bone invasion by oral squamous cell carcinoma. Oncotarget..

[33] Liu Y, Qi X, Li G, Sowa G (2019). Caveolin-2 deficiency induces a rapid anti-tumor immune response prior to regression of implanted murine lung carcinoma tumors. Sci Rep.

[34] Rojasawasthien T, Shirakawa T, Washio A, Tsujisawa T, Matsubara T, Inoue A (2021). Vignacyanidin polyphenols isolated from Vigna Angularis bean promote osteoblast differentiation. In Vivo.

[35] Miyawaki A, Rojasawasthien T, Hitomi S, Aoki Y, Urata M, Inoue A (2020). Oral administration of Geranylgeraniol rescues denervation-induced muscle atrophy via suppression of Atrogin-1. In Vivo.

[36] Shirakawa T, Miyawaki A, Matsubara T, Okumura N, Okamoto H, Nakai N, et al. Daily oral administration of protease-treated royal jelly protects against denervation-induced skeletal muscle atrophy. Nutrients. 2020;12:3089. 10.3390/nu12103089 PMC7600733 33050588

[37] Nath S, Devi GR (2016). Three-dimensional culture systems in cancer research: focus on tumor spheroid model. Pharmacol Ther.

[38] Rötzer V, Breit A, Waschke J, Spindler V (2014). Adducin is required for desmosomal cohesion in keratinocytes. J Biol Chem.

[39] Prechova M, Adamova Z, Schweizer A-L, Maninova M, Bauer A, Kah D, et al. Plectin-mediated cytoskeletal crosstalk controls cell tension and cohesion in epithelial sheets. J Cell Biol. 2022;221:e202105146. 10.1083/jcb.202105146 PMC8932528 35139142

[40] Matsubara T, Kokabu S, Nakatomi C, Kinbara M, Maeda T, Yoshizawa M (2018). The actin-binding protein PPP1r18 regulates maturation, actin organization, and bone resorption activity of osteoclasts. Mol Cell Biol.

[41] Matsubara T, Addison WN, Kokabu S, Neff L, Horne W, Gori F, et al. Characterization of unique functionalities in c-Src domains required for osteoclast podosome belt formation. J Biol Chem. 2021;296:100790. 10.1016/j.jbc.2021.100790 PMC8196221 34019873

[42] Talantov D, Mazumder A, Yu JX, Briggs T, Jiang Y, Backus J (2005). Novel genes associated with malignant melanoma but not benign melanocytic lesions. Clin Cancer Res.

[43] Gache Y, Chavanas S, Lacour JP, Wiche G, Owaribe K, Meneguzzi G (1996). Defective expression of plectin/HD1 in epidermolysis bullosa simplex with muscular dystrophy. J Clin Invest.

[44] Shimizu H, Masunaga T, Kurihara Y, Owaribe K, Wiche G, Pulkkinen L (1999). Expression of plectin and HD1 epitopes in patients with epidermolysis bullosa simplex associated with muscular dystrophy. Arch Derm Res.

[45] Winter L, Wiche G (2013). The many faces of plectin and plectinopathies: pathology and mechanisms. Acta Neuropathol.

[46] Gregor M, Osmanagic-Myers S, Burgstaller G, Wolfram M, Fischer I, Walko G (2014). Mechanosensing through focal adhesion-anchored intermediate filaments. FASEB J.

[47] Stover DR, Liebetanz J, Lydon NB (1994). Cdc2-mediated modulation of pp60c-src activity. J Biol Chem.

[48] Resnick RJ, Taylor SJ, Lin Q, Shalloway D (1997). Phosphorylation of the Src substrate Sam68 by Cdc2 during mitosis. Oncogene..

[49] Bruzzaniti A, Neff L, Sanjay A, Horne WC, De Camilli P, Baron R (2005). Dynamin forms a Src kinase-sensitive complex with Cbl and regulates podosomes and osteoclast activity. Mol Biol Cell.

[50] Ory S, Brazier H, Pawlak G, Blangy A (2008). Rho GTPases in osteoclasts: orchestrators of podosome arrangement. Eur J Cell Biol.

[51] Guarino M (2010). Src signaling in cancer invasion. J Cell Physiol.

[52] Burgstaller G, Gregor M, Winter L, Wiche G (2010). Keeping the vimentin network under control: cell–matrix adhesion–associated plectin 1f affects cell shape and polarity of fibroblasts. Mol Biol Cell.

[53] Yang C-Y, Chang P-W, Hsu W-H, Chang H-C, Chen C-L, Lai C-C (2019). Src and SHP2 coordinately regulate the dynamics and organization of vimentin filaments during cell migration. Oncogene..

[54] Matsuyoshi N, Hamaguchi M, Taniguchi S, Nagafuchi A, Tsukita S, Takeichi M (1992). Cadherin-mediated cell-cell adhesion is perturbed by v-src tyrosine phosphorylation in metastatic fibroblasts. J Cell Biol.

[55] Rötzer V, Hartlieb E, Vielmuth F, Gliem M, Spindler V, Waschke J (2015). E-cadherin and Src associate with extradesmosomal Dsg3 and modulate desmosome assembly and adhesion. Cell Mol Life Sci.

[56] Osmanagic-Myers S, Rus S, Wolfram M, Brunner D, Goldmann WH, Bonakdar N (2015). Plectin reinforces vascular integrity by mediating crosstalk between the vimentin and the actin networks. J Cell Sci.

[57] Jirouskova M, Nepomucka K, Oyman-Eyrilmez G, Kalendova A, Havelkova H, Sarnova L (2018). Plectin controls biliary tree architecture and stability in cholestasis. J Hepatol.

[58] Krausova A, Buresova P, Sarnova L, Oyman-Eyrilmez G, Skarda J, Wohl P (2021). Plectin ensures intestinal epithelial integrity and protects colon against colitis. Mucosal Immunol.

[59] Smalley KSM (2003). A pivotal role for ERK in the oncogenic behaviour of malignant melanoma?. Int J Cancer.

[60] Estrada Y, Dong J, Ossowski L (2009). Positive crosstalk between ERK and p38 in melanoma stimulates migration and in vivo proliferation. Pigment Cell Melanoma Res.

[61] Destaing O, Block MR, Planus E, Albiges-Rizo C (2011). Invadosome regulation by adhesion signaling. Curr Opin Cell Biol.

[62] Sutoh Yoneyama M, Hatakeyama S, Habuchi T, Inoue T, Nakamura T, Funyu T (2014). Vimentin intermediate filament and plectin provide a scaffold for invadopodia, facilitating cancer cell invasion and extravasation for metastasis. Eur J Cell Biol.

