# Supplementary material for: Plectin promotes tumor formation by B16 mouse melanoma cells via regulation of Rous sarcoma oncogene activity
Source: BMC Cancer. 2022 Aug 30;22:936. doi: 10.1186/s12885-022-10033-4 (PMC9426213; doi:10.1186/s12885-022-10033-4)
Supplement: Supplementary file 1 — Additional file 1: Fig S1. Generation of plectin knockdown human melanoma cells. Fig S2. Tumor weights and volumes. Fig S3. Cell proliferation is decreased in plectin knockdown human melanoma cells. Fig S4. Cell adhesion is impaired in plectin knock downed human melanoma cells. Fig S5. Vimentin was localized around the nucleus following Src activation in PKO cells. Fig S6. Plectin knockout decreased MAPK signaling. Fig S7. Expression of plectin and SRC-related genes are increased in human primary melanoma samples. [file 12885_2022_10033_MOESM1_ESM.pdf]

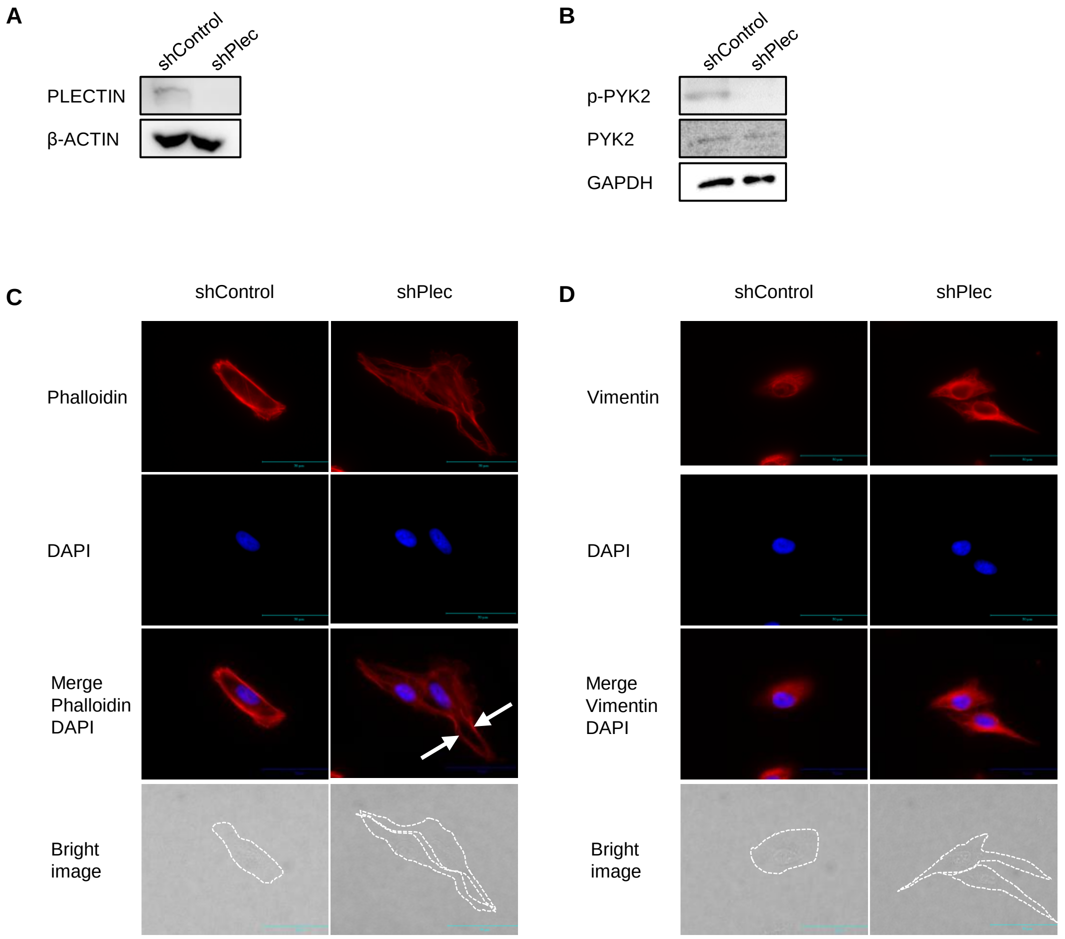

Fig S1. Generation of plectin knockdown human melanoma cells.

HMV-II cells were stably transfected with control (shControl) or plectin (shPlec) targeted shRNA. (A) Expression level of PLECTIN was determined by western blotting analysis. (B) Expression and phosphorylation of PYK2 or GAPDH was determined by western blotting analysis 24 h after plating. Full uncropped blots are shown in Fig S8. (C) Cells were immunostained with Rhodamine-phalloidin and DAPI. ells were immunostained with Rhodamine-phalloidin and DAPI. Cell shapes are outlined with a dotted line in the bright-field image. Scale bar = 50 µm. (D) Cells were immunostained with anti-vimentin/anti-mouse Alexa fluor 556 and DAPI. Scale bar = 50 µm.

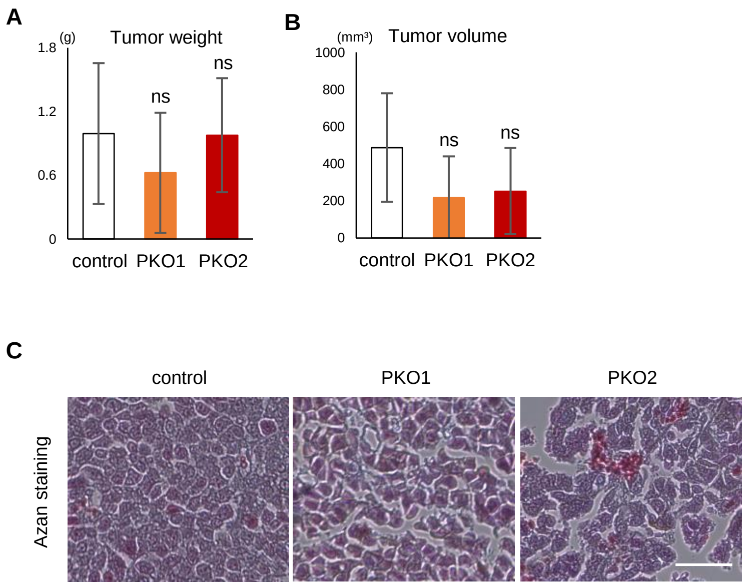

Fig S2. Tumor weights and volumes.

Control or PKO cells (1×10^5 cells per mouse) were subcutaneously injected into BALB/cAJcl-nu/nu mice (n = 8). Tumor weight (A) and volume (B) of each tumor was measured. (ns; no significance vs control) (C) Images of tumor sections stained with Azan stain.

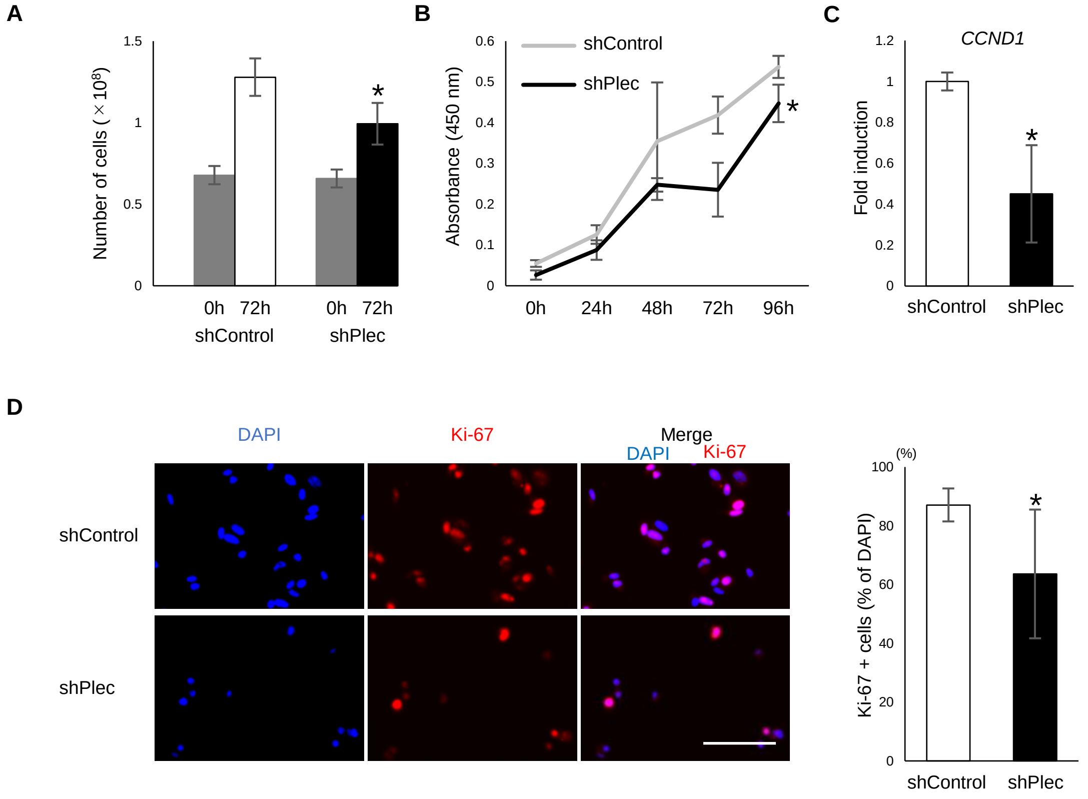

Fig S3. Cell proliferation is decreased in plectin knockdown human melanoma cells.
(A) Cells were plated a density of 2.5×10^4 cells per well in 24-well plates and the number of cells was counted by Cell viability assay (n = 3, *; $p < 0.05$ vs shControl). (B) The number of viable plectin knock downed HMV-II cells was determined by CCK-8 assay (n = 3, *; $p < 0.05$ vs shControl). (C) Expression of *CCND1* in plectin knock downed HMV-II cells was determined by real time qPCR (n = 3, *; $p < 0.05$ vs shControl). (D) HMV-II Cells were fixed and stained with anti-Ki-67/Alexa fluor 488 and DAPI at 1 d after plating. The percentage of Ki-67 positive cells relative to DAPI was calculated and indicated in the graph. Scale bar = 50 μm. (n = 5, *; $p < 0.05$ vs control).

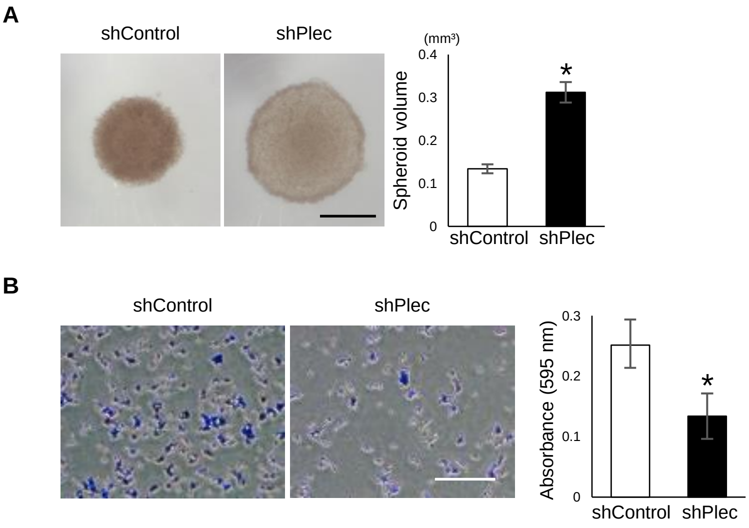

Fig S4. **Cell adhesion is impaired in plectin knock downed human melanoma cells.**
(A) Plectin knock downed HMV-II cells (5×10^3 cells per 15 µl drop) were cultured on a dish lid to form spheroid for 7 d. They were measured by ImageJ. Scale bar = 500 µm (n = 5, *; $p < 0.05$ vs shControl). (B) Cells were plated on a fibronectin-coated dish and counted the number of adherent cells after 4 hours. The adherent cells were stained with crystal violet (left 2 panels) and counted (right panel). scale bar =50µm (n = 6, *; $p < 0.05$ vs shControl).

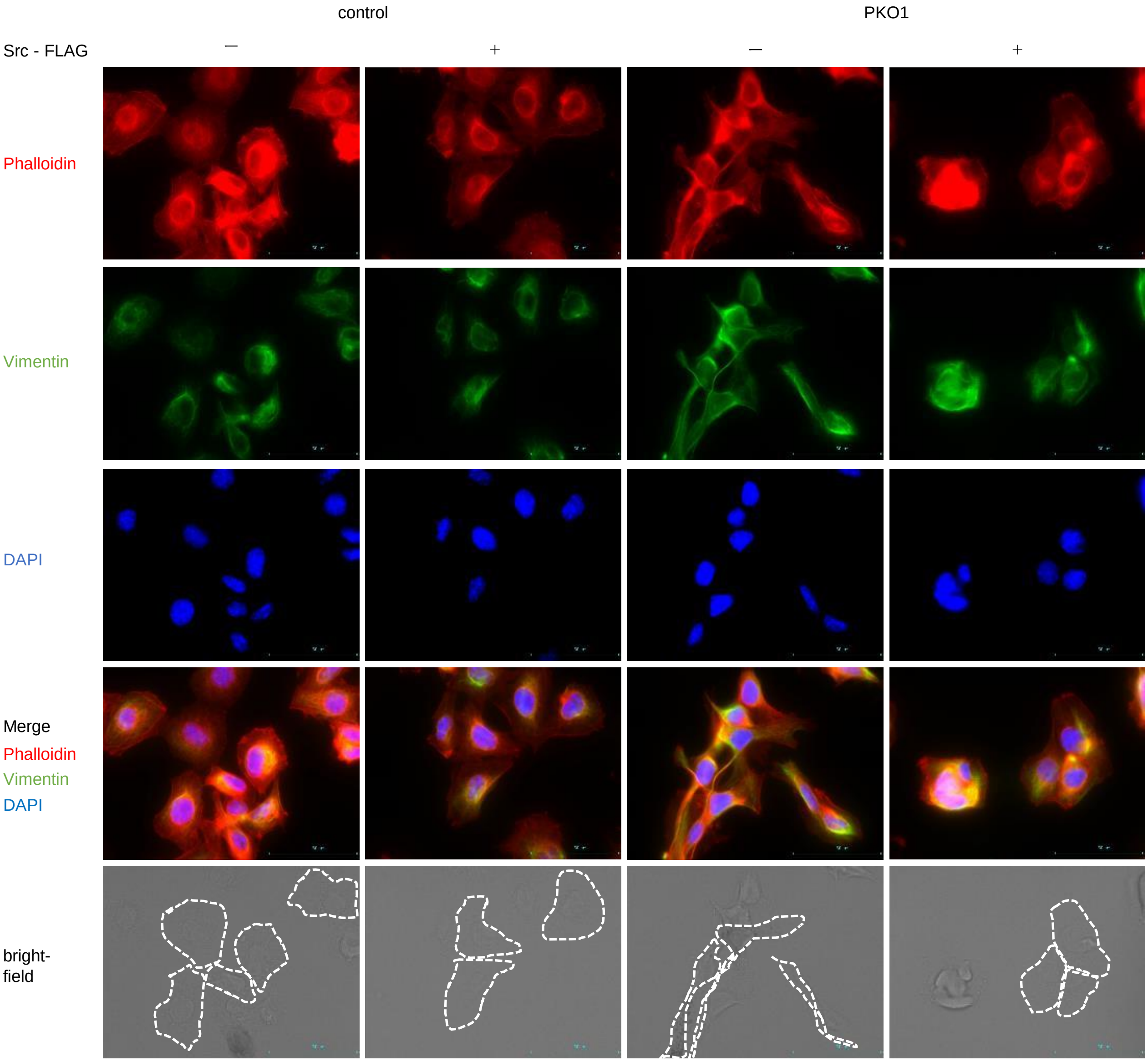

Fig S5. Vimentin was localized around the nucleus following Src activation in PKO cells. Cells were fixed and immunostained with Rhodamine-phalloidin, Vimentin/Alexa fluor 488 and DAPI at 2 d after constitutively activated Src introduction. Scale bar = 50 μm.

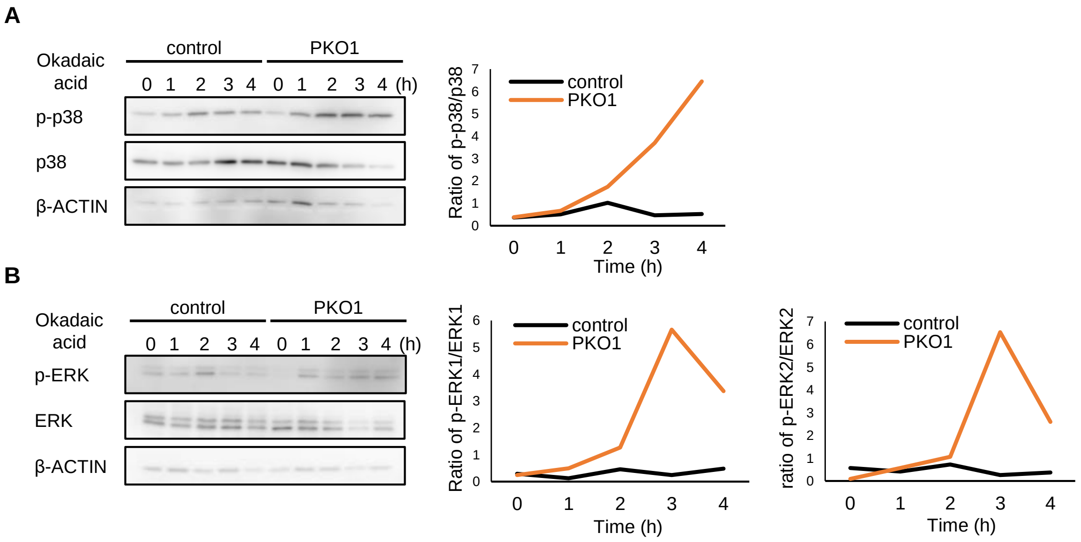

Fig S6. Plectin knockout decreased MAPK signaling.

Cell lysate were prepared at the indicated time points following okadaic acid treatment. Expression and phosphorylation of (A) p38 or phosphorylation of (B) ERK were determined by western blotting analysis. The expression level was measured with ImageJ.

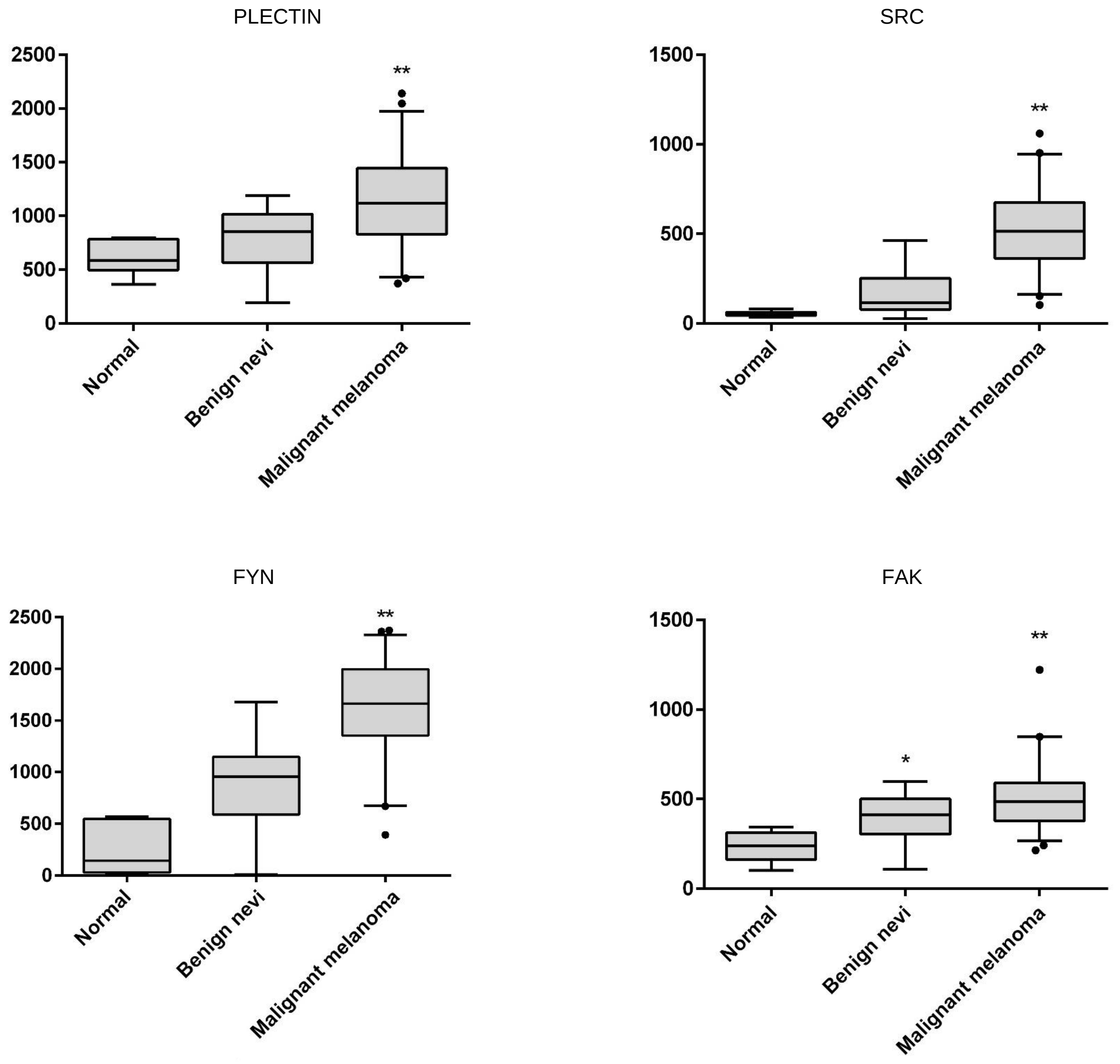

Fig S7. Expression of plectin and SRC-related genes are increased in human primary melanoma samples.

mRNA levels of *PLEC*, *SRC*, *FYN* and *FAK* in normal skin, benign nevi and melanoma of patients in GSE1375 dataset. Statistical significance of differences between groups were analyzed using Kruskal-Wallis followed by Dunn’s post hoc test. (*; $p < 0.05$ vs normal tissue, **; $p < 0.01$ vs benign skin nevi).
