# Supplementary material for: Plectin promotes tumor formation by B16 mouse melanoma cells via regulation of Rous sarcoma oncogene activity
Source: BMC Cancer. 2022 Aug 30;22:936. doi: 10.1186/s12885-022-10033-4 (PMC9426213; doi:10.1186/s12885-022-10033-4)
Supplement: Supplementary file 2 — Additional file 2. [file 12885_2022_10033_MOESM2_ESM.pdf]

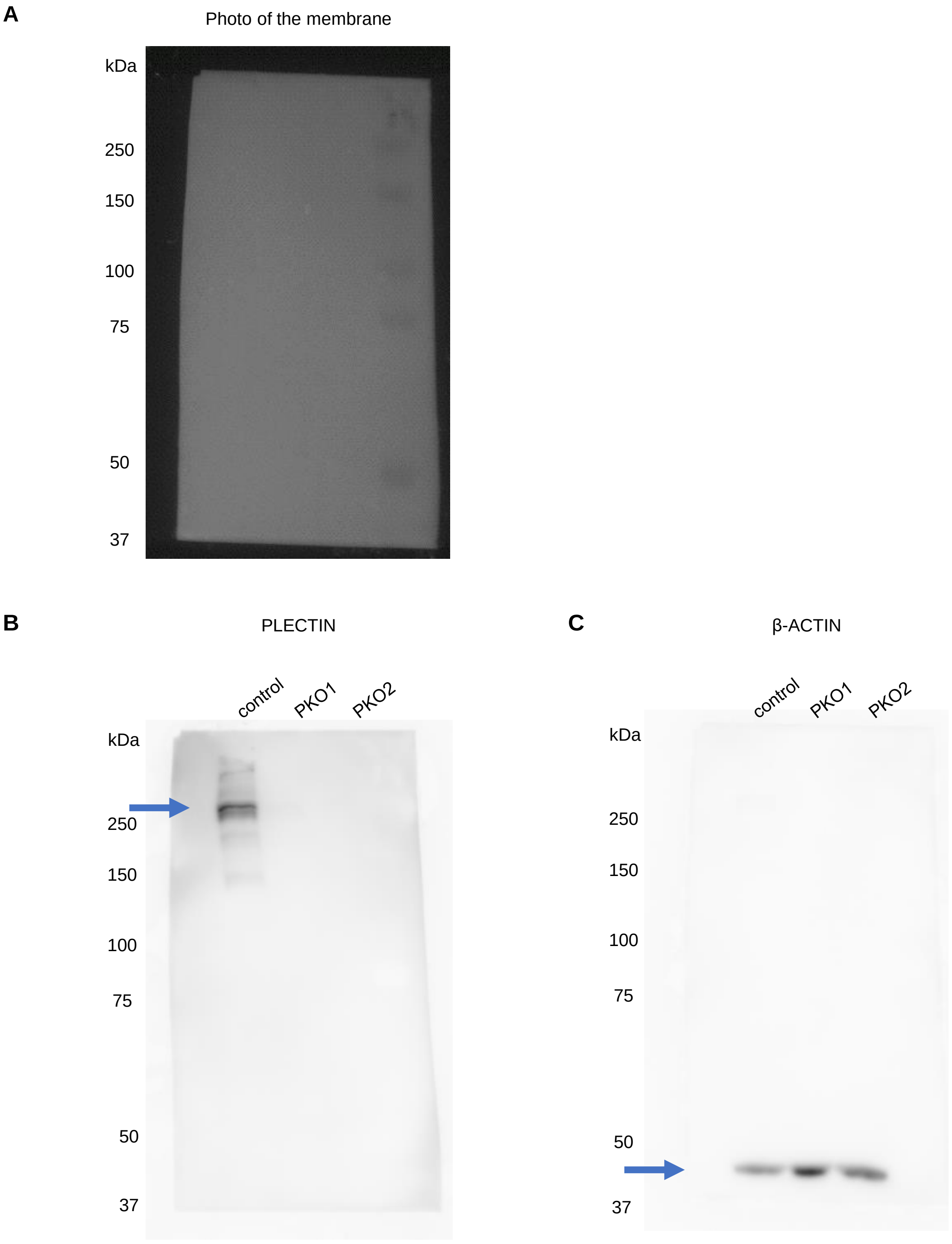

Full uncropped blots of Figure 1A
(A) Photo of the membrane for western blotting analysis. (B) PLECTIN is blotted about 500 kDa indicated by the arrow. (C) β-ACTIN is blotted about 42 kDa indicated by the arrow.

A

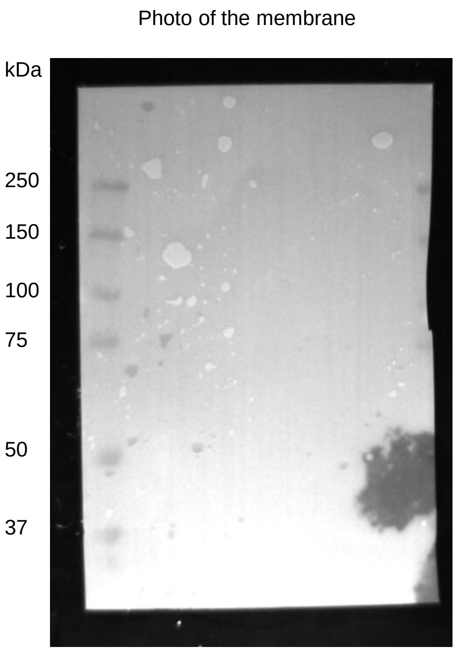

B

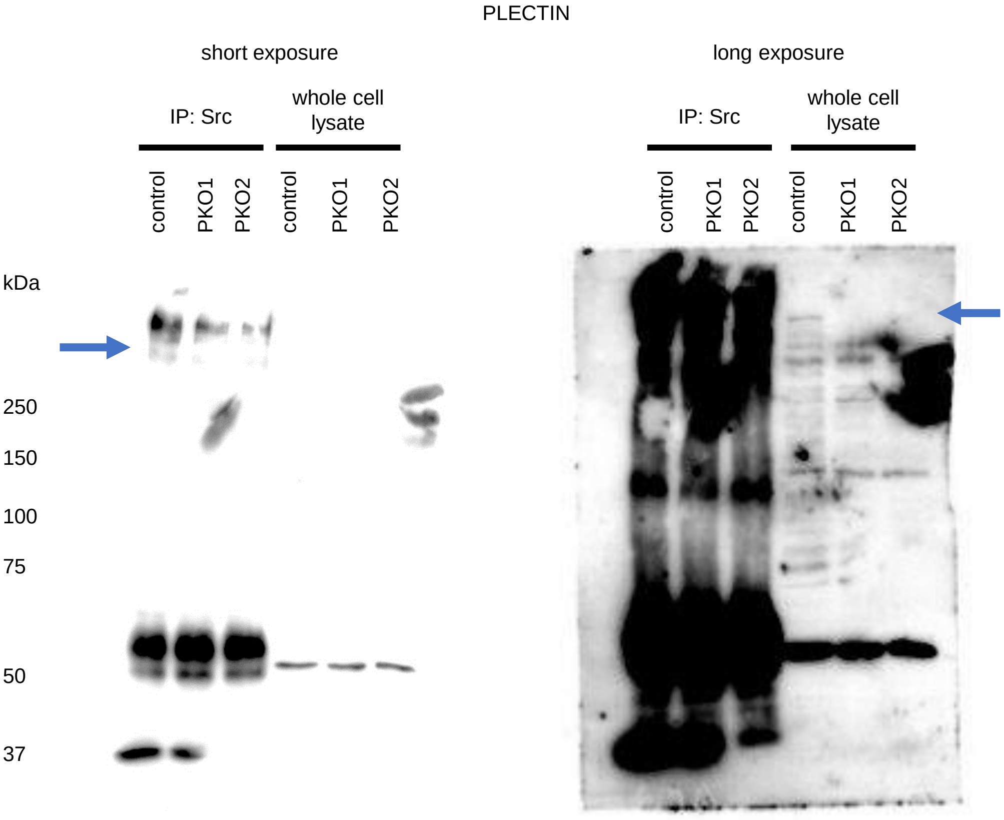

Full uncropped blots of Figure 1B

(A) Photo of the membrane for western blotting analysis. (B) PLECTIN is blotted about 500 kDa indicated by the arrow. Short exposure image (left) and long exposure image (right) are shown.

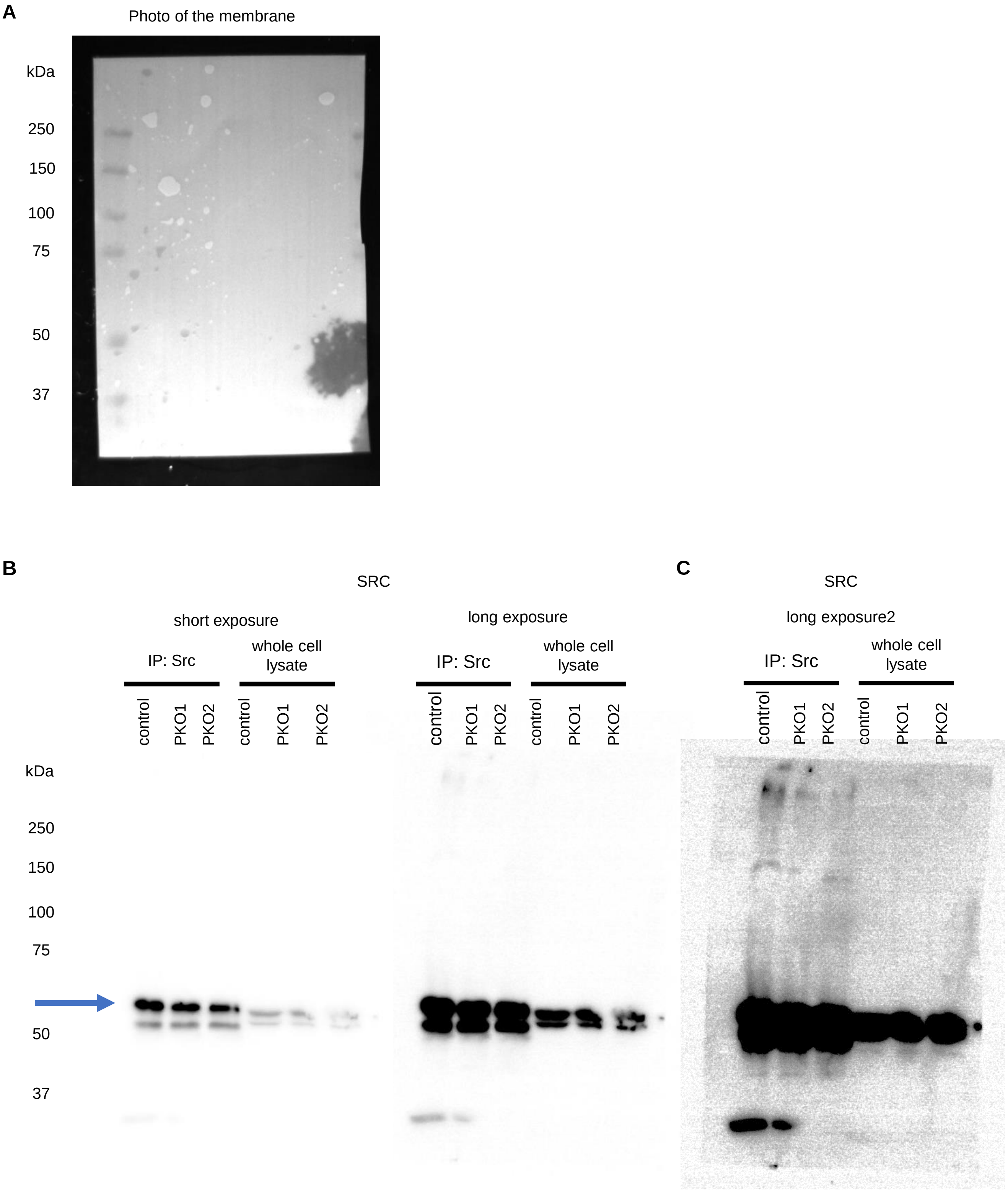

Full uncropped blots of Figure 1B

SRC is re-blotted after PLECTIN blotting. (A) Photo of the membrane for western blotting analysis. (B) SRC is blotted about 60 kDa indicated by the arrow. Short exposure image (left) and long exposure image (right) are shown. (C) More long-time exposure image than (B) is shown to certify the membrane.

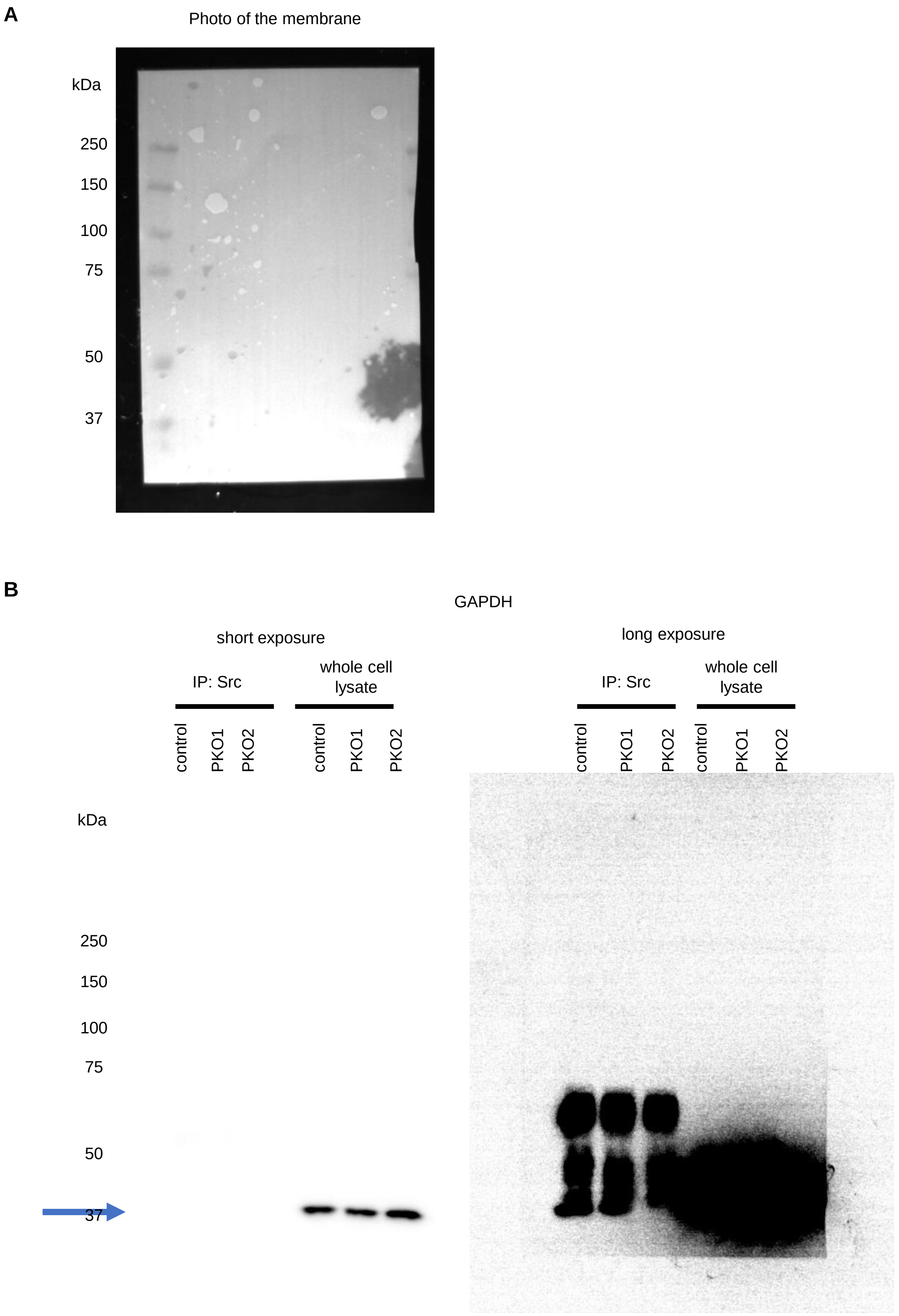

Full uncropped blots of Figure 1B

GAPDH is re-blotted after PLECTIN and SRC blotting. (A) Photo of the membrane for western blotting analysis. (B) GAPDH is blotted about 38 kDa indicated by the arrow. Short exposure image (left) and long exposure image (right) are shown.

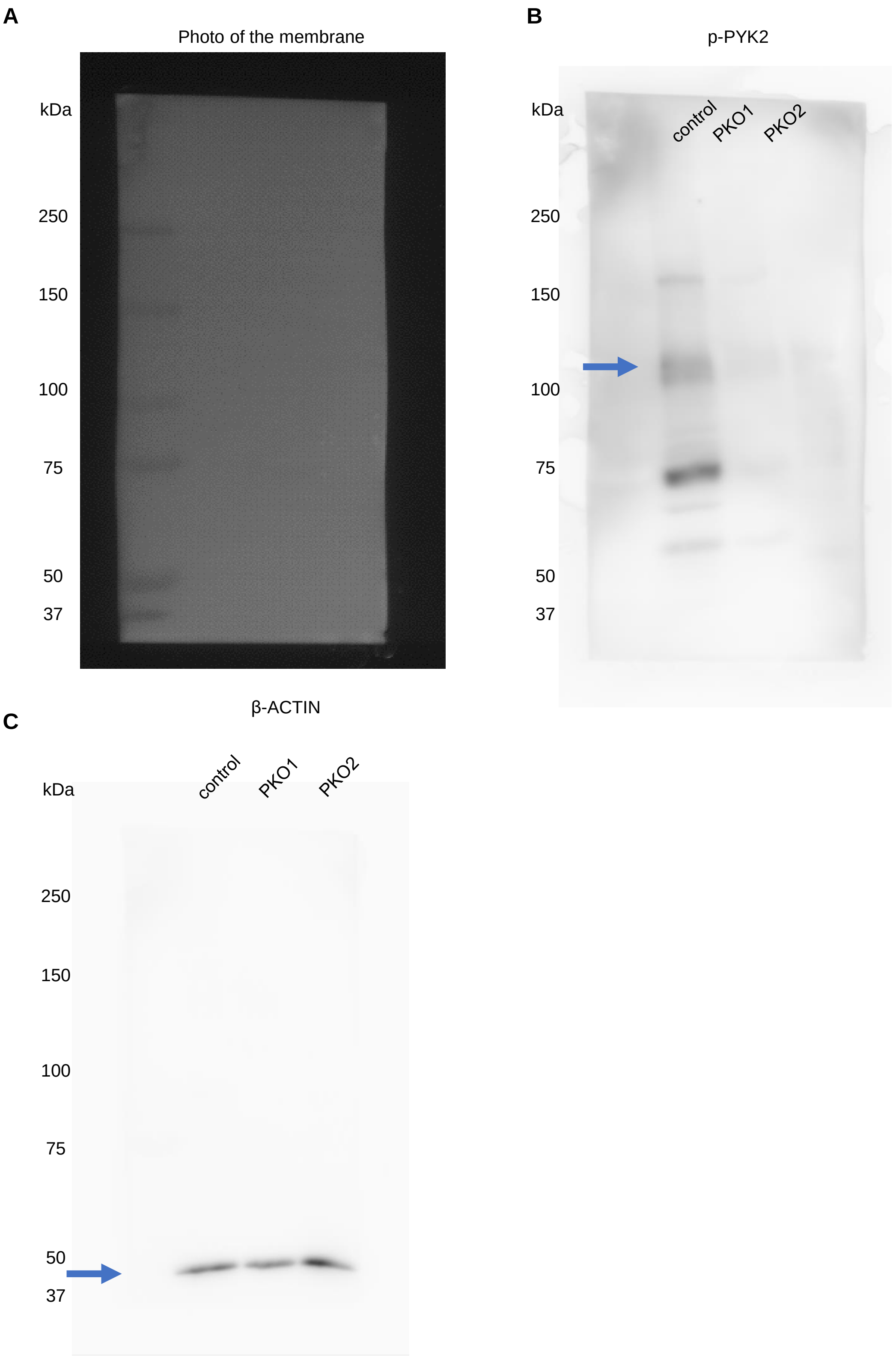

Full uncropped blots of Figure 1C

(A) Photo of the membrane for western blotting analysis. (B) p-PYK2 is blotted about 120 kDa indicated by the arrow. (C) β-ACTIN is blotted about 42 kDa indicated by the arrow. β-ACTIN is re-blotted after p-PYK2 blotting.

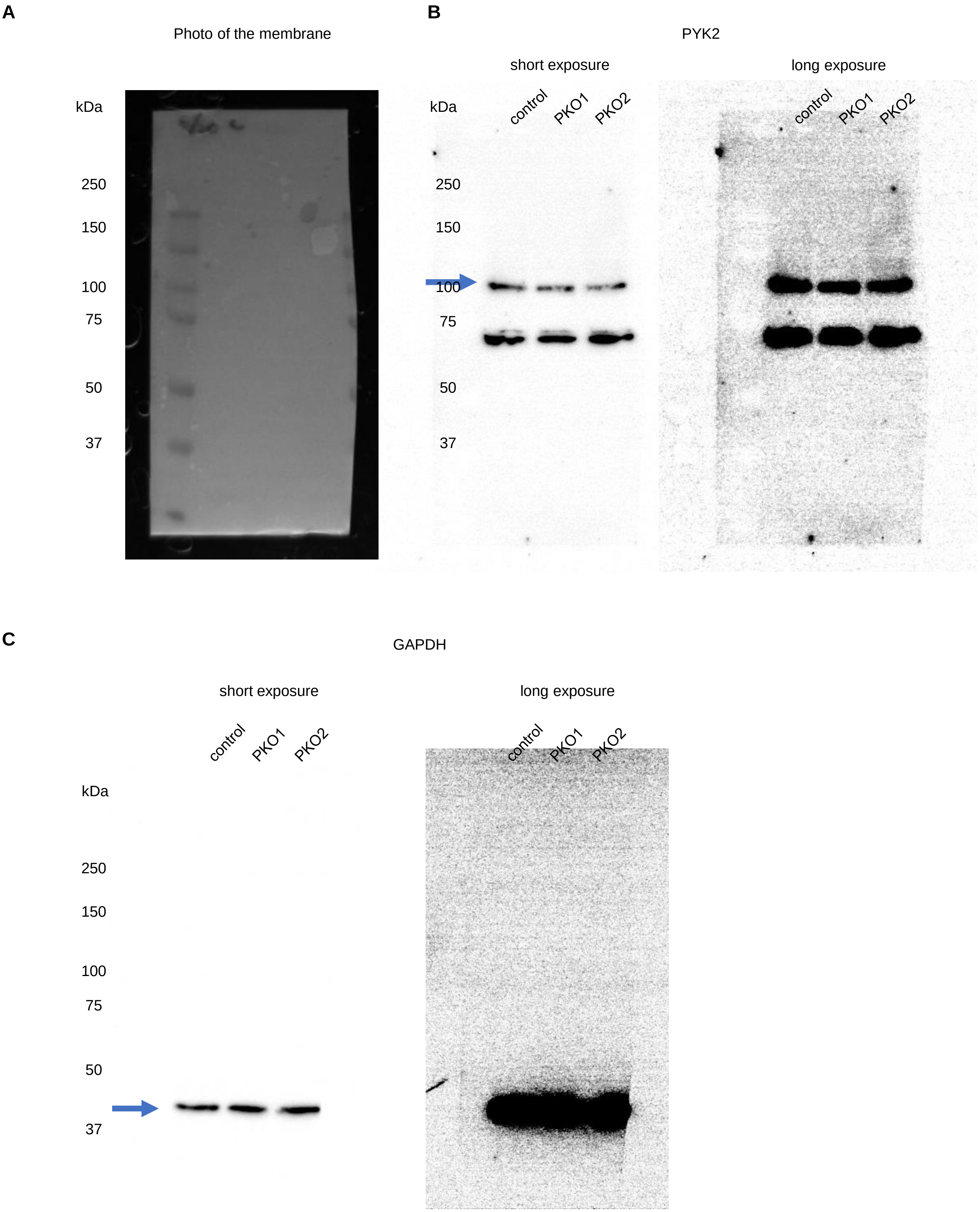

Full uncropped blots of Figure 1C

Same lysates that examined p-PYK were loaded.

(A) Photo of the membrane for western blotting analysis. (B) PYK2 is blotted about 120 kDa indicated by the arrow. Short exposure image (left) and long exposure image (right) are shown. (C) GAPDH is blotted about 38 kDa indicated by the arrow. Short exposure image (left) and long exposure image (right) are shown. GAPDH is re-blotted after PYK2 blotting.

A

Photo of the membrane

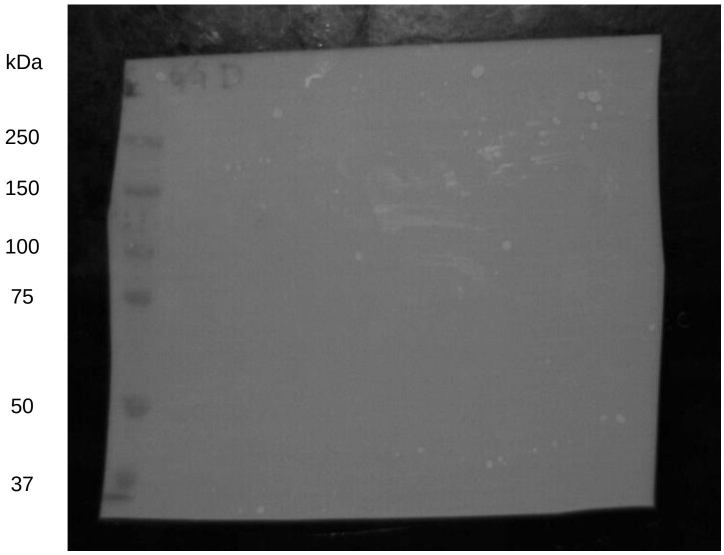

B

PYK2

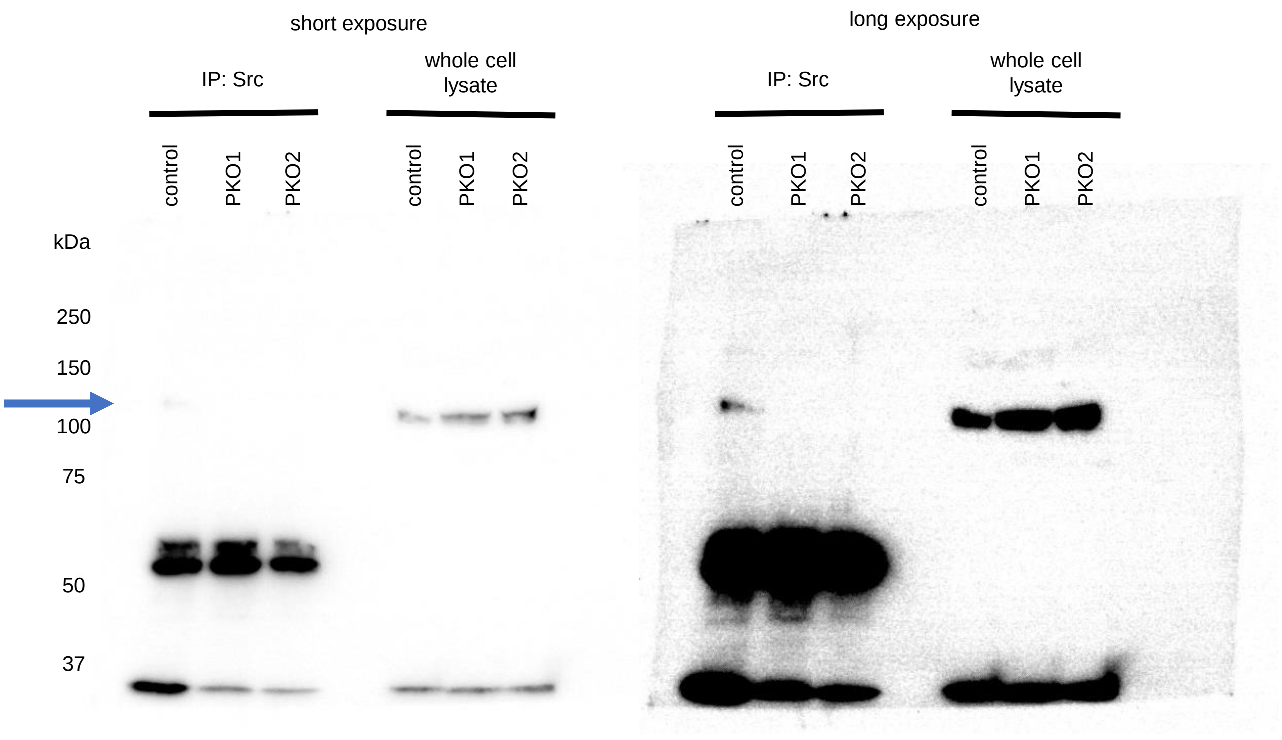

Full uncropped blots of Figure 1D

(A) Photo of the membrane for western blotting analysis. (B) PYK2 is blotted about 120 kDa indicated by the arrow. Short exposure image (left) and long exposure image (right) are shown.

A

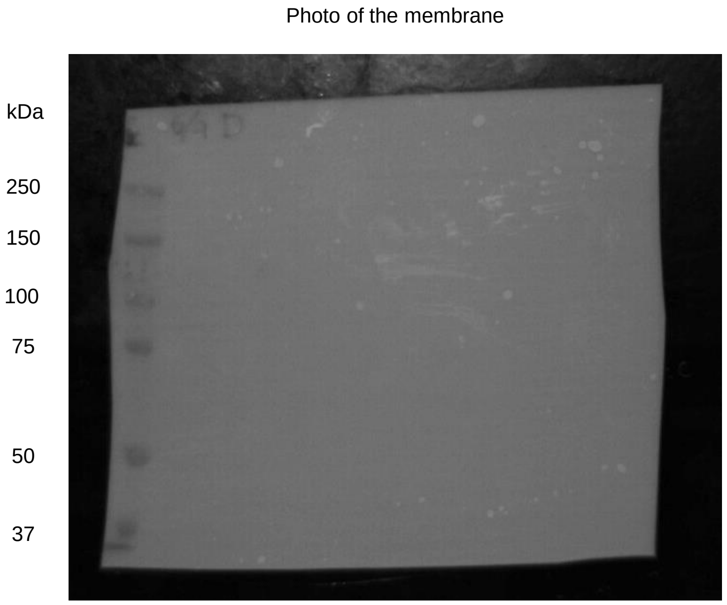

B

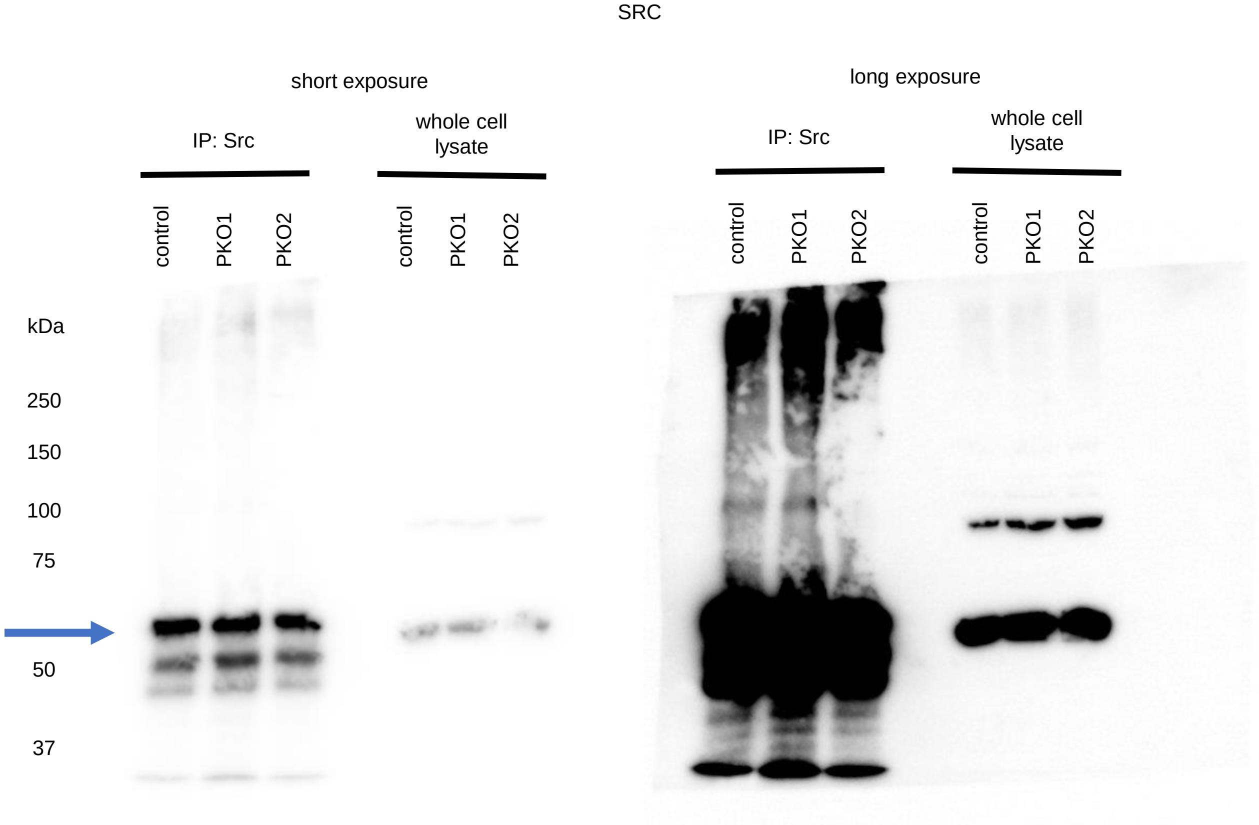

Full uncropped blots of Figure 1D

SRC is re-blotted after PYK2 blotting. (A) Photo of the membrane for western blotting analysis. (B) SRC is blotted about 60 kDa indicated by the arrow. Short exposure image (left) and long exposure image (right) are shown.

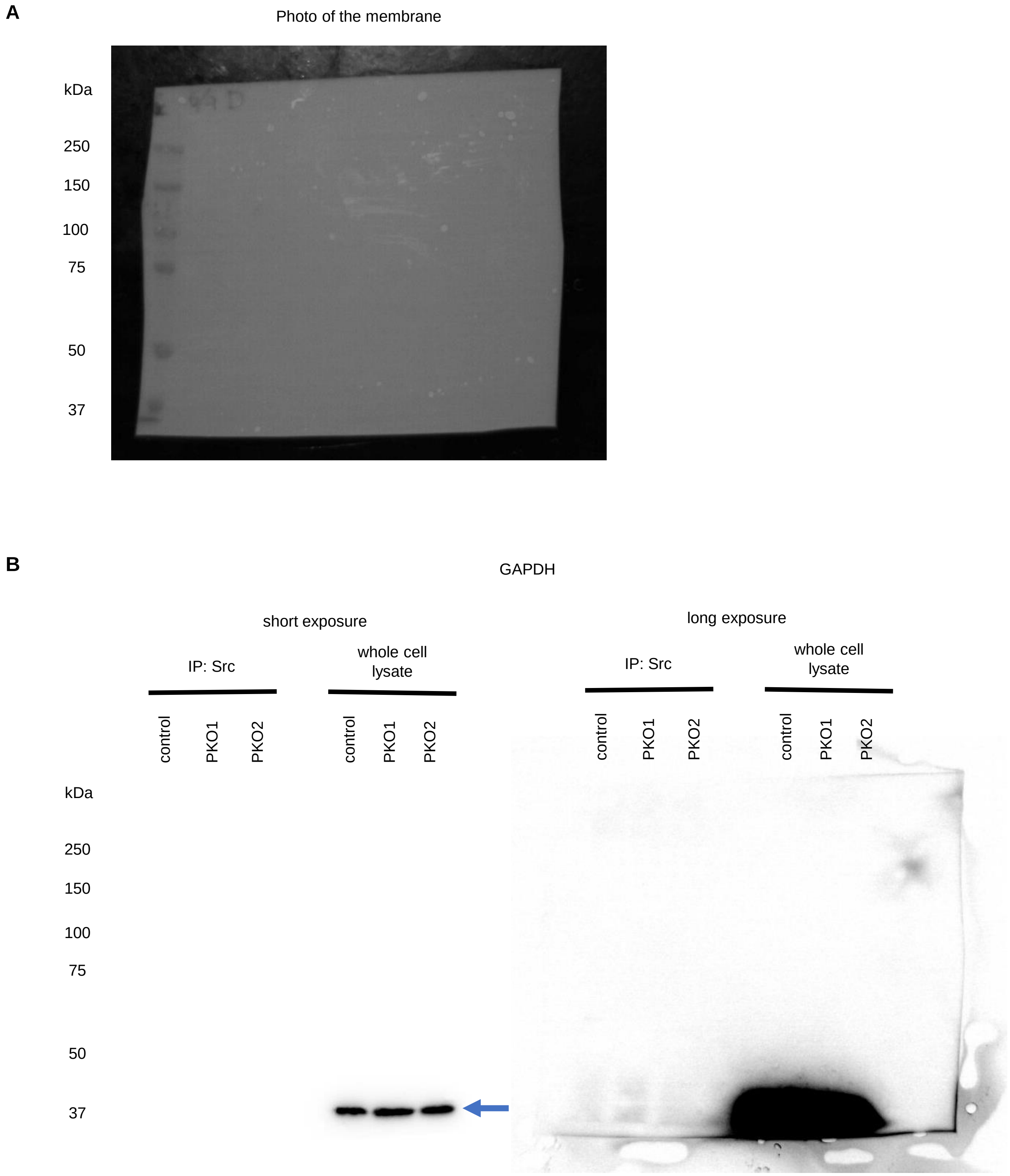

Full uncropped blots of Figure 1D

GAPDH is re-blotted after PLECTIN and SRC blotting. (A) Photo of the membrane for western blotting analysis. (B) GAPDH is blotted about 38 kDa indicated by the arrow. Short exposure image (left) and long exposure image (right) are shown.

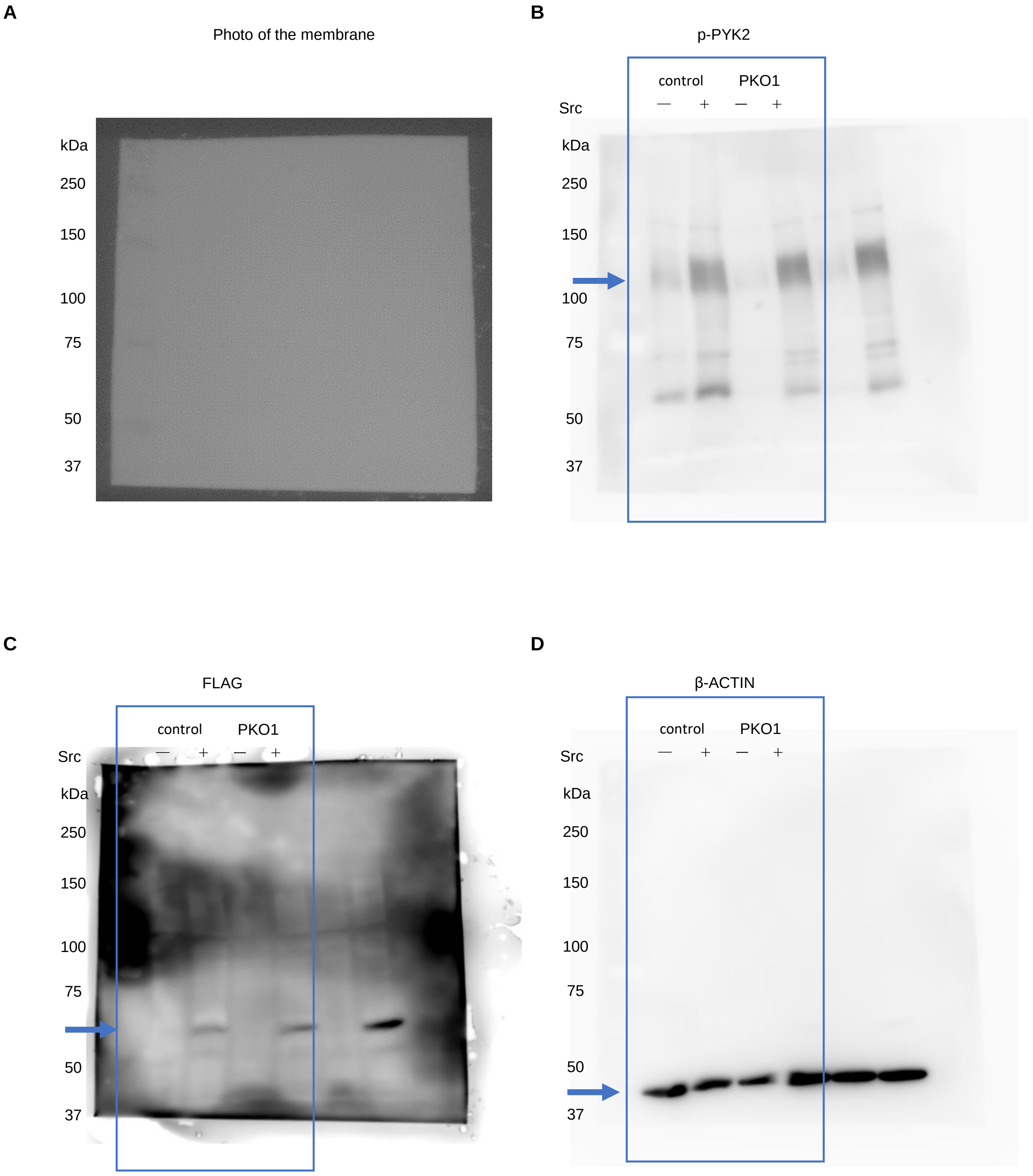

Full uncropped blots of Figure 5A

(A) Photo of the membrane for western blotting analysis. (B) p-PYK2 is blotted about 120 kDa indicated by the arrow. (C) FLAG is blotted about 60 kDa indicated by the arrow. FLAG is re-blotted after p-PYK2 blotting. (D) β-ACTIN is blotted about 42 kDa indicated by the arrow. β-ACTIN is re-blotted after p-PYK2 and FLAG blotting.

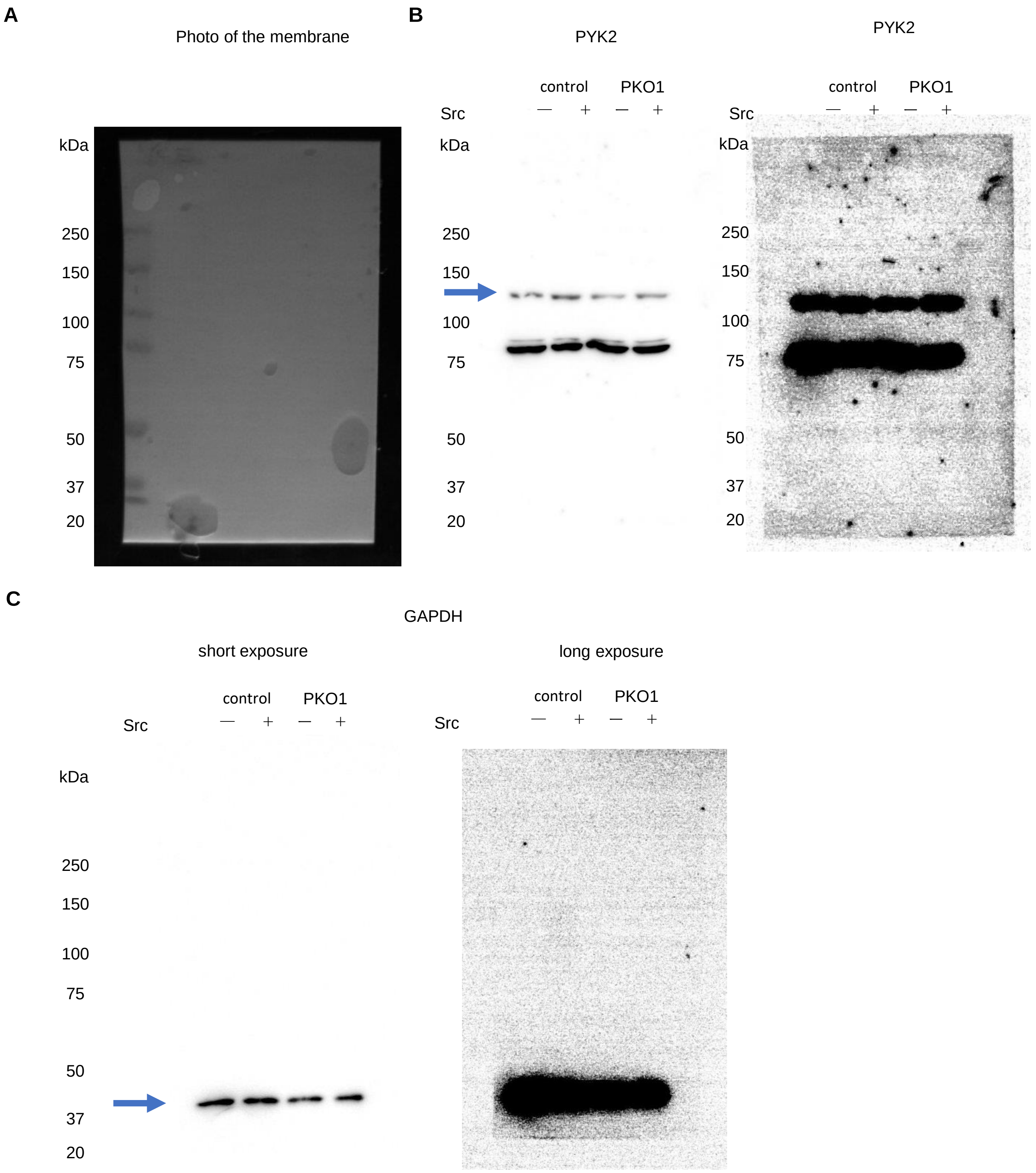

Full uncropped blots of Figure 5A

Same lysates that examined p-PYK were loaded.

(A) Photo of the membrane for western blotting analysis. (B) PYK2 is blotted about 120 kDa indicated by the arrow. Short exposure image (left) and long exposure image (right) are shown. (C) GAPDH is blotted about 38 kDa indicated by the arrow. Short exposure image (left) and long exposure image (right) are shown. GAPDH is re-blotted after PYK2 blotting.

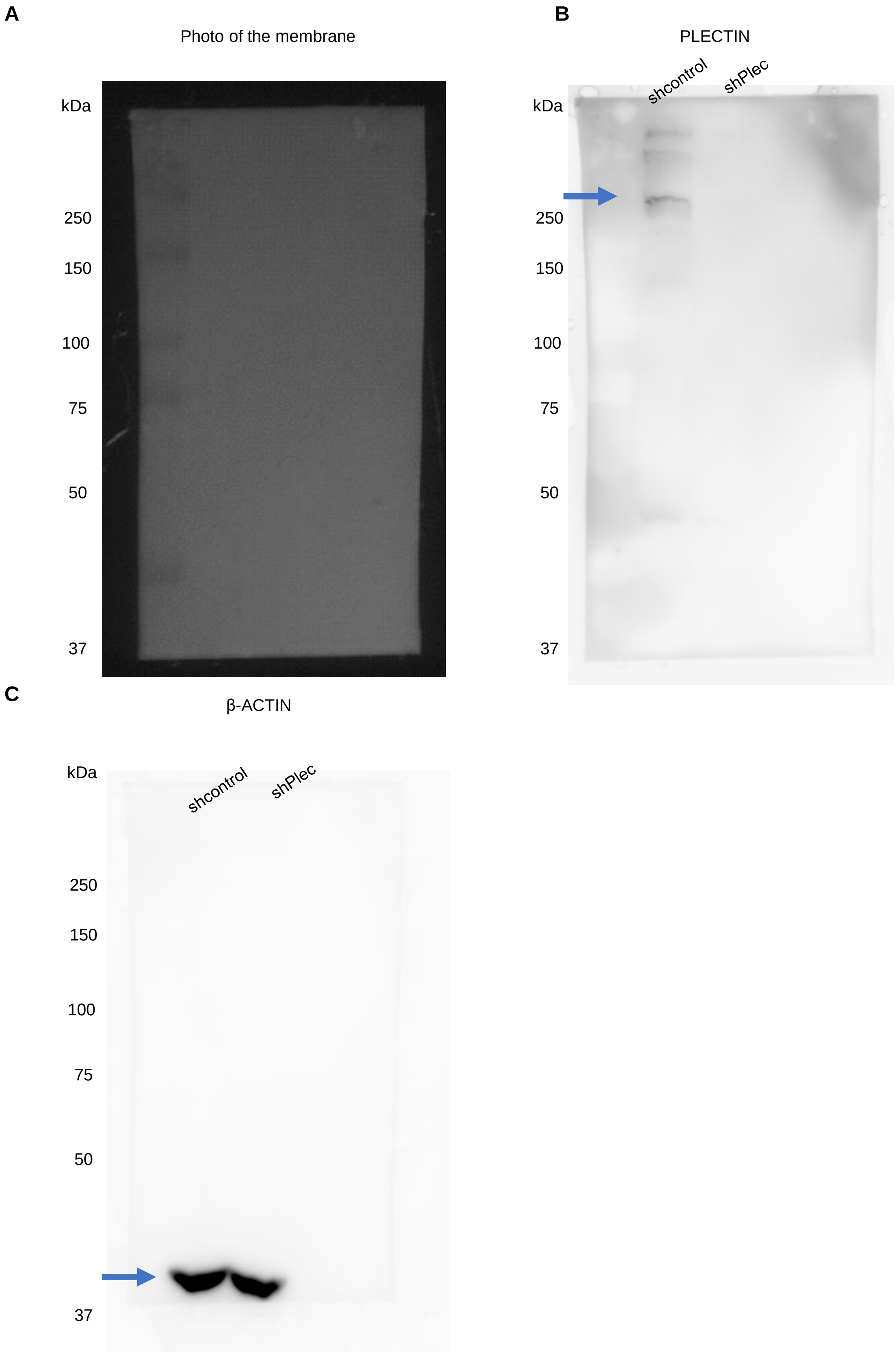

Full uncropped blots of Figure S1A

(A) Photo of the membrane for western blotting analysis. (B) PLECTIN is blotted about 500 kDa indicated by the arrow. (C) β-ACTIN is blotted about 42 kDa indicated by the arrow. β-ACTIN is re-blotted after PLECTIN blotting.

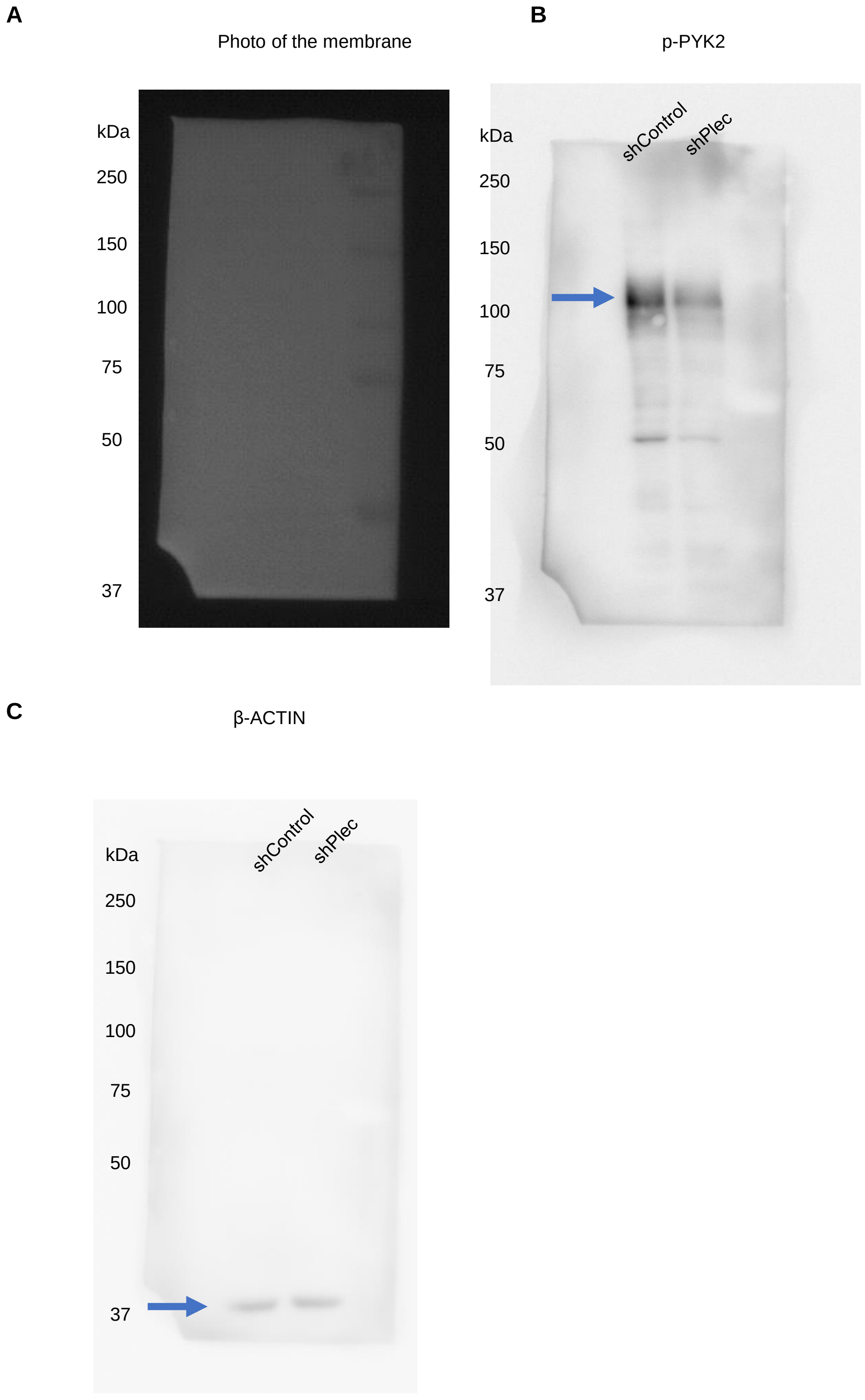

Full uncropped blots of Figure S1B

(A) Photo of the membrane for western blotting analysis. (B) p-PYK2 is blotted about 120 kDa indicated by the arrow. (C) β-ACTIN is blotted about 42 kDa indicated by the arrow. β-ACTIN is re-blotted after p-PYK2 blotting.

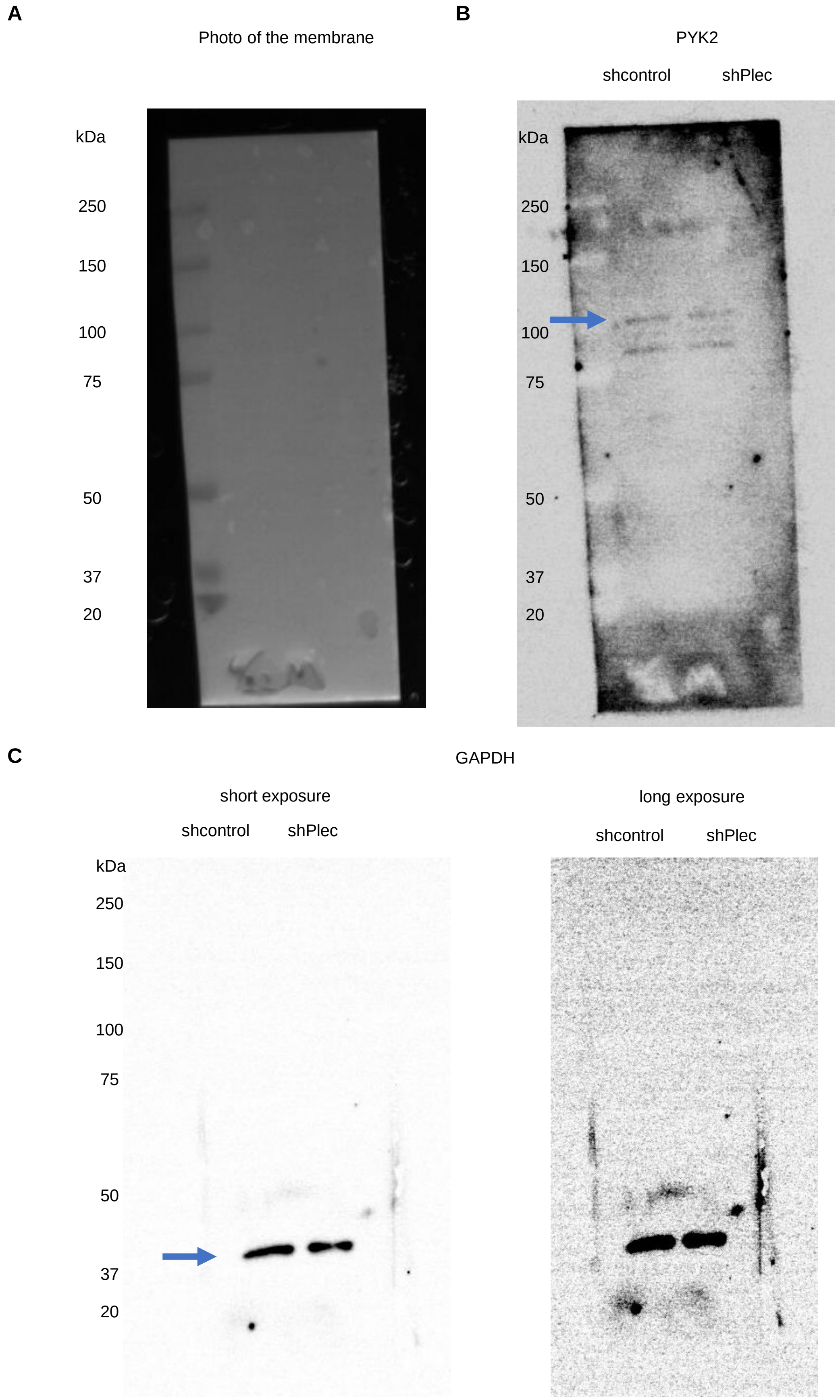

Full uncropped blots of Figure S1B

(A) Photo of the membrane for western blotting analysis. (B) PYK2 is blotted about 120 kDa indicated by the arrow. (C) GAPDH is blotted about 38 kDa indicated by the arrow. Short exposure image (left) and long exposure image (right) are shown. GAPDH is re-blotted after PYK2 blotting.

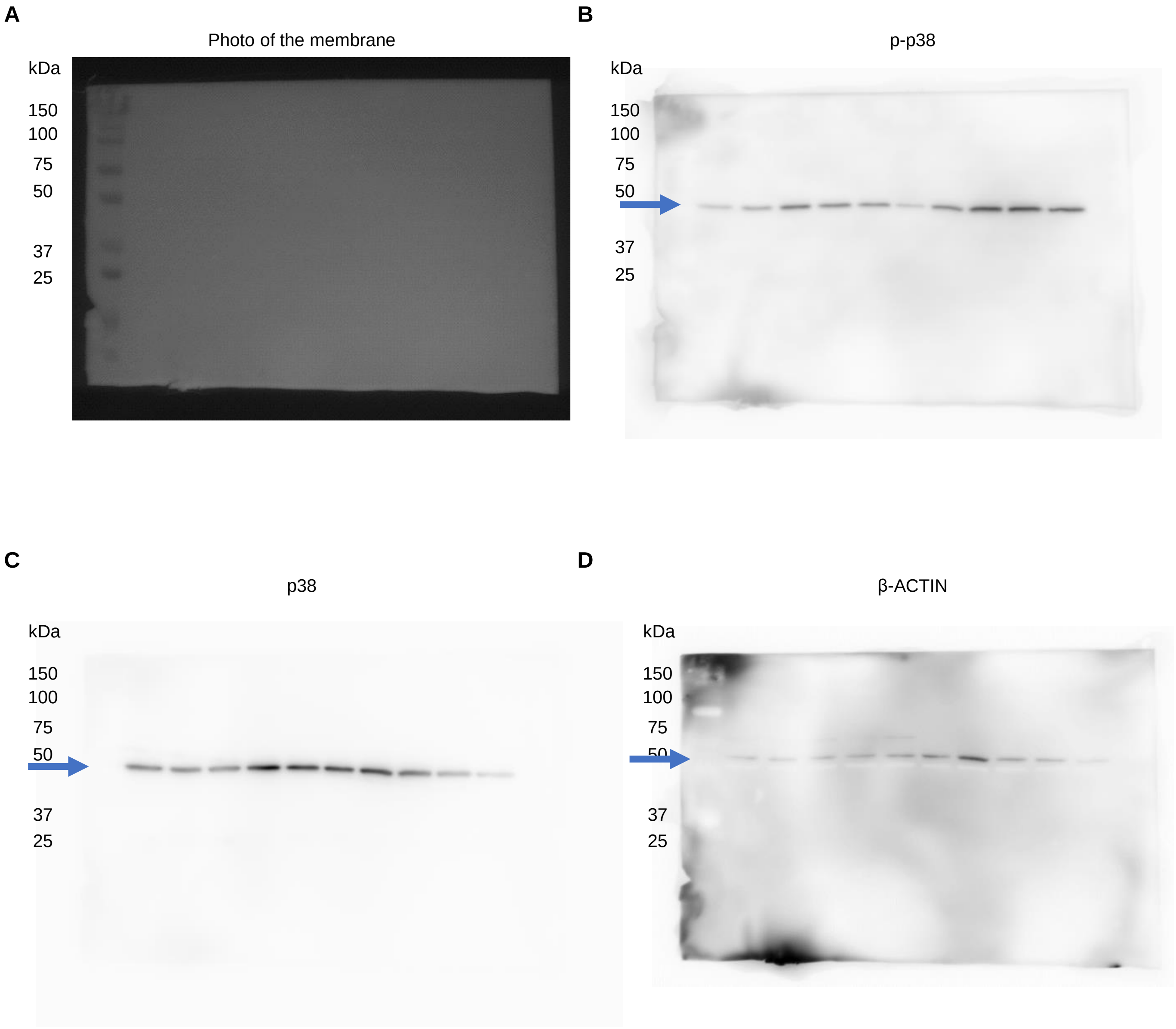

Full uncropped blots of Figure S6A

(A) Photo of the membrane for western blotting analysis. (B) p-p38 is blotted about 43 kDa indicated by the arrow . (C) p38 is blotted about 43 kDa indicated by the arrow. p38 is re-blotted after p-p38 blotting. (D)β-ACTIN is blotted about 42 kDa indicated by the arrow. β-ACTIN is re-blotted after p-p38 and p38 blotting.

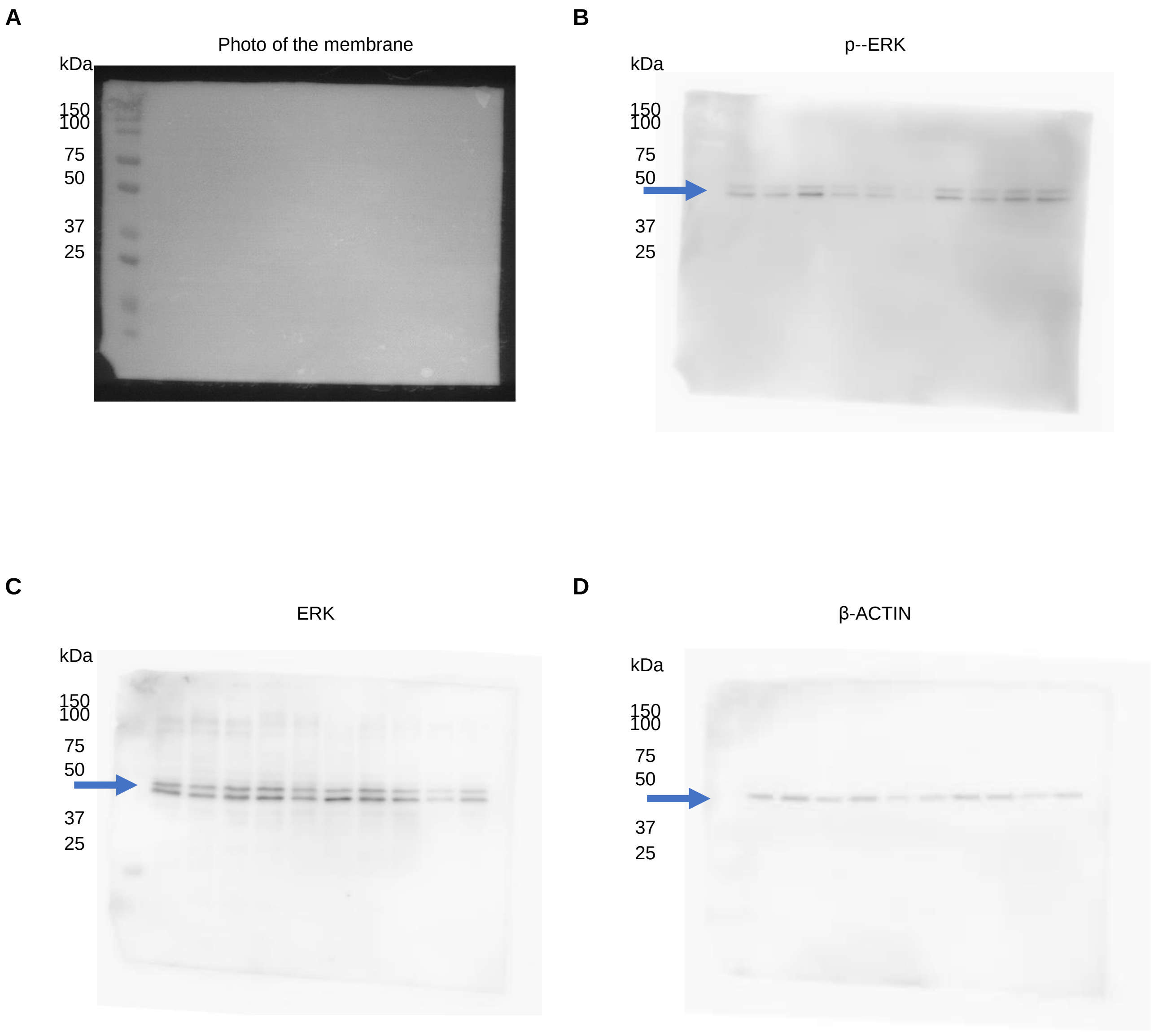

Full uncropped blots of Figure S6B

(A) Photo of the membrane for western blotting analysis. (B) p-ERK1/2 is blotted about 42kDa (Erk1), 44 kDa (Erk2) indicated by the arrow .

(C) ERK1/2 is blotted about 42, 44 kDa indicated by the arrow. ERK1/2 is re-blotted after p-ERK1/2 blotting. (D)β-ACTIN is blotted about 42 kDa indicated by the arrow. β-ACTIN is re-blotted after p-ERK1/2 and ERK1/2 blotting.
